# The innate immune sensor Toll-like receptor 2 controls the senescence-associated secretory phenotype

**DOI:** 10.1126/sciadv.aaw0254

**Published:** 2019-06-05

**Authors:** Priya Hari, Fraser R. Millar, Nuria Tarrats, Jodie Birch, Andrea Quintanilla, Curtis J. Rink, Irene Fernández-Duran, Morwenna Muir, Andrew J. Finch, Valerie G. Brunton, João F. Passos, Jennifer P. Morton, Luke Boulter, Juan Carlos Acosta

**Affiliations:** 1 Cancer Research UK Edinburgh Centre, Institute of Genetics and Molecular Medicine, University of Edinburgh, Edinburgh EH4 2XU, UK.; 2 Institute for Cell and Molecular Biosciences, Campus for Ageing and Vitality, Newcastle University Institute for Ageing, Newcastle University, Newcastle upon Tyne NE4 5PL, UK.; 3 Cancer Research UK Beatson Institute, Glasgow G61 1BD, UK.; 4 Institute of Cancer Sciences, University of Glasgow, Glasgow G61 1QH, UK.; 5 Department of Physiology and Biochemical Engineering Mayo Clinic, 200 First Street SW, Rochester, MN 55905, USA.; 6 MRC-Human Genetics Unit, Institute of Genetics and Molecular Medicine, University of Edinburgh, Edinburgh EH4 2XU, UK.

## Abstract

Cellular senescence is a stress response program characterized by a robust cell cycle arrest and the induction of a proinflammatory senescence-associated secretory phenotype (SASP) that is triggered through an unknown mechanism. Here, we show that, during oncogene-induced senescence (OIS), the Toll-like receptor 2 (TLR2) and its partner TLR10 are key mediators of senescence in vitro and in murine models. TLR2 promotes cell cycle arrest by regulating the tumor suppressors p53-p21^CIP1^, p16^INK4a^, and p15^INK4b^ and regulates the SASP through the induction of the acute-phase serum amyloids A1 and A2 (A-SAAs) that, in turn, function as the damage-associated molecular patterns (DAMPs) signaling through TLR2 in OIS. Last, we found evidence that the cGAS-STING cytosolic DNA sensing pathway primes TLR2 and A-SAAs expression in OIS. In summary, we report that innate immune sensing of senescence-associated DAMPs by TLR2 controls the SASP and reinforces the cell cycle arrest program in OIS.

## Teaser

Innate immune sensing is essential for the proinflammatory program in cellular senescence.

## INTRODUCTION

Cellular senescence is a cell cycle arrest program induced by various stresses that renders cells insensitive to mitogenic signals and impairs the proliferation and expansion of damaged cells (*1*). Activation of oncogenes such as *RAS* in somatic cells induces a senescence program termed oncogene-induced senescence (OIS) (*2*). OIS is a cell-intrinsic tumor suppressor mechanism that impairs tumor progression (*2*–*7*). Senescence is also characterized by the activation of the senescence-associated secretory phenotype (SASP). The SASP is a cocktail of proinflammatory cytokines, chemokines, growth factors, and matrix-remodeling proteins with diverse functions and roles (*8*–*10*). Through the SASP, senescent cells elicit multiple paracrine effects to promote the normal processes associated with inflammation, wound healing, tissue remodeling, and cell plasticity (*11*–*14*) or it can disrupt tissue homeostasis, promoting aging and other pathophysiological conditions such as fibrosis and cancer (*12*). The SASP reinforces the cell cycle arrest by activating p53 and the cell cycle inhibitors p21^CIP1^ and p15^INK4b^ (*8*, *10*, *15*). The SASP also promotes paracrine senescence (*15*) and stimulates immune surveillance that results in clearance of the senescent cells (*16*–*18*), which collectively contribute to the tumor suppressor program elicited by OIS. Therefore, the immune system can regulate senescence and determine senescent cell fate (*19*–*21*).

The activation of the SASP is primarily regulated by the transcription factors nuclear factor κB (NF-κB) and CCAAT/enhancer-binding protein β (C/EBPβ) (*8*, *10*, *22*). SASP activation responds to a hierarchical model where interleukin-1α (IL-1α) and IL-1β are the apical cytokines signaling through IL-1 receptor (IL-1R), triggering a cascade of additional amplifying feedback loops by other cytokines and chemokines of the SASP. As a consequence, NF-κB and C/EBPβ activation are sustained, and the expression of the SASP is maintained. Interfering with these feedback loops, such as the one set up by IL-1, IL-6, or IL-8, collapses the network, impairing the induction of the SASP and the activation of the cell cycle arrest (*8*, *10*, *15*, *23*).

We have previously shown that the inflammasome regulates the activation of the SASP (*15*). The inflammasome is a multiprotein platform that induces the proteolytic activity of the inflammatory cysteine-aspartic protease Caspase-1. It is a key regulatory component of the innate immune response and the first line of defense against pathogens and damage (*24*). Activation of the inflammasome leads to cleavage and activation of the proinflammatory cytokine IL-1β. Inflammasomes are controlled by a family of receptors called pattern recognition receptors (PRRs). PRRs are receptors of the innate immune system that are activated by interaction with pathogen-associated molecular patterns (PAMPs) or with damage-associated molecular patterns (DAMPs) that are generated endogenously in cells under certain conditions of stress and damage (*25*). There are three major PRR families: Toll-like receptors (TLRs), RIG-I–like receptors, and NOD-like receptors (*25*). Upon activation, PRRs induce distinct signal transduction pathways that activate an immune transcriptional program mostly regulated by NF-κB and interferon regulatory factors (IRFs).

Although the steady-state signaling of the SASP is relatively well understood, the molecular mechanism(s) that initiates the SASP in OIS and how the inflammasome is primed during OIS remain ill defined. Here, we describe the mechanism that underlies the priming of the inflammasome in OIS by PRRs, identifying the senescence-associated DAMP that initiates the SASP and reinforces OIS.

## RESULTS

### The innate immune receptor TLR2 is induced during cellular senescence

**Fig. 1:**
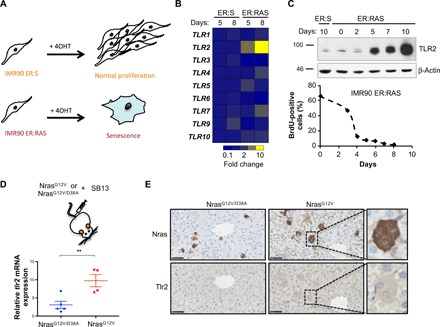
TLR2 expression is induced during OIS. (**A**) Schematic showing IMR90 ER:RAS cells treated with 4OHT undergo OIS. IMR90 ER:STOP cells serve as a control and retain proliferative capacity with 4OHT. (**B**) Quantitative reverse transcription polymerase chain reaction (qRT-PCR) analysis of TLR family member expression in IMR90 ER:RAS and ER:STOP cells treated with 4OHT for 5 and 8 days. (**C**) Western blot of TLR2 expression in IMR90 ER:RAS and ER:STOP (ER:S) cells with up to 10 days of 4OHT treatment (top). The 5-bromo-2′-deoxyuridine (BrdU) incorporation in IMR90 ER:RAS cells treated with 4OHT for up to 8 days, as indicated. (**D**) RNA was extracted from snap-frozen liver samples from wild-type (WT) mice 6 days following hydrodynamic delivery of Nras^G12V/D38A^ (*n* = 5) and Nras^G12V^ (*n* = 4) transposons. *tlr2* mRNA expression was measured using qRT-PCR. Scatter plots represents value per animal, with the horizontal line representing group means ± SEM. Statistical significance was calculated using Students two-tailed *t* test. **P < 0.01. (**E**) Immunohistochemical staining for Nras and Tlr2 in consecutive liver sections from corresponding mice in (D) showing that oncogenic Nras^G12V^ expressing, but not Nras^G12V/D38A^ expressing, hepatocytes express Tlr2. Scale bars, 50 μm.

To study OIS, we used the well-characterized IMR90 human diploid fibroblast cell line transduced with an Estrogen Receptor:H-RAS^G12V^ fusion protein (henceforth referred to as ER:RAS). ER:RAS oncogenic activity is induced after addition of 4-hydroxytamoxifen (4OHT) to the cultures, resulting in a time-controlled OIS response as previously described (*15*) (Fig. 1A). Gene set enrichment analysis (GSEA) of the transcriptome of IMR90 ER:RAS senescent cells showed an enrichment for “innate immunity,” “PRR,” and “TLR” terms (fig. S1A). Therefore, we decided to explore the role of TLRs in OIS. Analysis of the gene expression of all 10 human TLR genes showed a marked induction of *TLR2* expression in OIS (Fig. 1B and fig. S1, B and C). The induction of *TLR2* mRNA correlated with an increase in TLR2 protein (Fig. 1C), which corresponded with the cell cycle arrest during OIS (Fig. 1C). The induction of *TLR2* was also observed after additional OIS by retroviral transduction of oncogenic H-Ras^G12V^, by the activation of senescence with a DNA damage-inducing agent (etoposide), and by conditioned medium from OIS cells (paracrine senescence) (fig. S1, B to E) (*15*). However, the induction of senescence with nongenotoxic agents such as the cyclin-dependent kinase 4 (CDK4) inhibitor palbociclib or the mouse double minute 2 homolog (MDM2) inhibitor Nutlin 3a failed to induce *TLR2* and the SASP (fig. S1, B and C), suggesting that the activation of TLR2 expression is associated to genotoxic stress.

In vivo, we analyzed *tlr2* expression in four well-characterized mouse models of senescence. We first examined the expression of *tlr2* in a murine model of OIS, in which conditional expression of Kras^G12D^ by Pdx-CRE (KC mouse) induces pancreatic intraepithelial neoplasia (PanIN) (*26*). A marked induction of Tlr2 was observed in ductal pancreatic cells in PanINs with low Ki67 indexes, indicating that Tlr2 is expressed in early senescent PanINs (fig. S2A). We then investigated an additional model where OIS is induced in murine hepatocytes via hydrodynamic delivery of a mutant Nras^G12V^ encoding transposon along with a sleeping beauty transposase–expressing plasmid (*16*). A plasmid encoding an Nras^G12V^ effector loop mutant, incapable of downstream Nras signaling (Nras^G12V/D38A^), was used as a negative control. Six days after Nras^G12V^ transduction, *tlr2* mRNA expression was significantly increased in comparison to controls (Fig. 1D), which correlated with the expected induction of mRNA expression of the senescence markers *dcr2* and *arf* and the SASP factor IL-1β (fig. S2B). To investigate whether *tlr2* induction in this model derives from Nras-expressing hepatocytes or recruited immune cells, we performed immunohistochemistry (IHC) for Nras and Tlr2 protein expressions in consecutive liver sections. Analysis of these sections revealed that the expression of Tlr2 was induced in hepatocytes of mice transduced with active Nras and was not present in the inactive control (Fig. 1E). Moreover, superposition of consecutive sections stained for Nras and Tlr2 showed an overlap between both signals, suggesting that the hepatocytes expressing active Nras^G12V^ are indeed the cells overexpressing Tlr2 (Fig. 1E and fig. S2C). We then investigated the expression of *tlr2* in a model of inflammatory-mediated senescence, where knockout of *nfkb1* (*nfkb1^−/−^)* in mice leads to constitutive activation of NF-κB (*27*). An increased number of airway epithelial cells expressing Tlr2 were detected, which correlated with p21^Cip1^ expression in the lung airways (fig. S2D). Last, we observed an increase in the number of cells positively expressing Tlr2 in alveolar cells of the lung in aged mice (fig. S2E). These results indicate that TLR2 is induced in cellular senescence.

### TLR2 controls the activation of the SASP in OIS

**Fig. 2:**
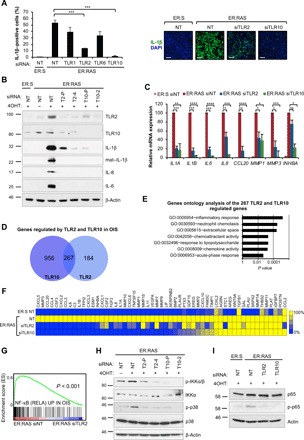
TLR2 and TLR10 regulate the SASP in OIS. (**A**) Immunofluorescence staining and high-content analysis for IL-1β expression in IMR90 ER:RAS cells treated with 4OHT for 8 days and repeatedly transfected with pooled siRNA targeting TLR1, TLR2, TLR6, and TLR10. Nontarget (NT)-pooled siRNA was used as control. Representative images are shown. Scale bars, 250 μm. DAPI, 4′,6-diamidino-2-phenylindole. (**B**) Western blot analysis against indicated antibodies in IMR90 ER:RAS cells treated with 4OHT for 8 days and repeatedly transfected with pooled and individual siRNA targeting TLR2 and TLR10. T2-P, siRNA TLR2 pool; T2-4, individual TLR2 siRNA; T10-P, siRNA TLR10 pool; T10-2, individual TLR10 siRNA. Nontarget pooled siRNA was used as control. Western blot against β-actin is shown as a loading control. (**C**) SASP factor regulation by qRT-PCR in ER:RAS cells treated with 4OHT for 8 days and repeatedly transfected with pooled siRNA targeting TLR2 and TLR10. Results are expressed as means ± SEM of three independent experiments. (**D**) Venn diagram showing the number of genes that are significantly induced by TLR2 and TLR10 during OIS in the transcriptome analysis (AmpliSeq) in IMR90 ER:RAS cells treated with 4OHT and transfected with pooled siRNA targeting TLR2 and TLR10 for 8 days (GSE127116). The intersection represents the number of genes regulated by both TLR2 and TLR10. This signature of 267 genes will be used for GSEA in additional senescence transcriptomes in Fig. 4 and figs. S4 and S7. (**E**) Top regulated terms identified through of coregulated genes in (H) using DAVID gene ontology analysis. Chart bars represent Benjamin-adjusted *P* value of term enrichment. (**F**) Heat map of SASP factor expression obtained from the transcriptome analysis (AmpliSeq) in IMR90 ER:RAS cells following siRNA knockdown of TLR2 and TLR10 for 8 days of 4OHT treatment. (**G**) GSEA enrichment plot of RELA signature in TLR2 siRNA-transfected IMR90 ER:RAS 4OHT-induced cells. (**H**) IMR90 ER:RAS cells were transfected with indicated siRNA for 8 days with 4OHT. Western blot for phosphorylation and total levels of IKKa/β and p38 mitogen-activated protein kinase (MAPK) was performed. (**I**) IMR90 ER:RAS cells were treated with 4OHT and repeatedly transfected with indicated pooled siRNA and nontarget siRNA as control for 5 days. Western blots were conducted for phosphorylation of p65 and total p65 protein levels. All statistical significance was calculated using one-way analysis of variance (ANOVA). ***P < 0.001, **P < 0.01, and *P < 0.05. ns, not significant.

We previously identified that activation of IL-1R signaling by the inflammasome was essential for the induction of the SASP (*15*). However, before inflammasome activation, the expression of IL-1β should be primed (*28*, *29*). TLR2 forms functional heterodimers with three other TLRs: TLR1, TLR6, and TLR10 (*30*–*32*). To evaluate the activity of all four members of the TLR2 network in IL-1β priming, we knocked down their expression during OIS using four pooled small interfering RNAs (siRNAs) (fig. S3A). While knockdown of TLR1 and TLR6 marginally decreased the expression of IL-1β, TLR2 and TLR10 knockdown strongly decreased its induction (Fig. 2, A to C, and fig. S3B), suggesting a predominant role for these receptors in priming the inflammasome in OIS. To rule out off-target effects from the siRNAs, we tested all four individual siRNAs from the TLR2 and TLR10 pool. Each siRNA produced efficient TLR2 knockdown in IMR90 ER:RAS cells (fig. S3C) and decreased the expression of IL-1β in OIS (fig. S3D). Similarly, two single siRNAs from the pooled siRNA also efficiently reduced the expression of TLR10 and impaired IL-1β transcriptional activation (fig. S3, C and E). TLR2 and TLR10 knockdown resulted in reduced production of mature active IL-1β (Fig. 2B) and a decrease in the accumulation of mature IL-1β in conditioned media of IMR90 ER:RAS cells (fig. S3B), indicating a crucial role for the TLR2 network for the inflammasome function in senescence. Inflammasome activation is a key step in the induction of the SASP during OIS (*15*). Therefore, we studied the effect of TLR2 signaling in SASP induction. Targeting TLR2 and TLR10 with siRNA impaired the induction of mRNA expression of the SASP components IL-1α, IL-6, IL-8, CCL20, matrix metalloproteinase 1 (MMP1), MMP3, and inhibin beta A (INHBA) (Fig. 2C) and blocked the induction of IL-8 and IL-6 proteins (Fig. 2B and fig. S3F). Moreover, inhibition of TLR2 with oxidized-1-palmitoyl-2-arachidonyl-sn-glycero-3-phosphorylcholine (OxPAPC) significantly reduced IL-1α and IL-1β mRNA induction in OIS (fig. S3G). Last, knockdown of TLR2 and TLR10 in IMR90 cells impaired the induction of IL-1β, IL-6, and IL-8 when exposed to conditioned media from senescent IMR90 ER:RAS cells, indicating a contribution of TLR2 in the propagation of the SASP during paracrine senescence (fig. S3H).

We then performed a transcriptomic analysis of more than 20,000 genes of IMR90 ER:RAS cells transfected with siRNA targeting TLR2 and TLR10 (fig. S3I). We found that TLR2 knockdown significantly regulated more than 1000 genes, while TLR10 knockdown regulated up to 2500 genes (fig. S3I and data file S1). From the total number of genes positively regulated by TLR2 and TLR10 in OIS, 267 were common targets, indicating a high degree of overlap between genes positively regulated by TLR2 and TLR10 (Fig. 2D and data file S2). The vast majority of genes regulated by TLR10 in OIS (956) were also not controlled by TLR2 in these experimental conditions (Fig. 2D and data file S3). Gene ontology analysis of these genes indicated a significant enrichment in “nucleotide binding proteins”–related terms (fig. S3J). In contrast, gene ontology analysis of the genes commonly regulated by TLR2 and TLR10 in OIS showed enrichment for terms related to “inflammatory response,” “chemotaxis,” “chemokine activity,” and “extracellular space” (Fig. 2E). Moreover, the analysis revealed that TLR2 and TLR10 regulated the expression of most of the proinflammatory SASP, most notably chemokines, cytokines, and metalloproteases (Fig. 2F). TLR2 signals through NF-κB and p38 mitogen-activated protein kinase (MAPK) (*33*), which are key SASP regulators. A significant enrichment in genes regulated by NF-κB in OIS (*22*) was observed in the transcriptome of control cells compared to TLR2-depleted cells (Fig. 2G), indicating an essential role for TLR2 signaling in NF-κB activation in senescence. To confirm this, we targeted TLR2 and TLR10 with siRNA in 4OHT-treated IMR90 ER:RAS cells and showed a decrease in NF-κB pathway activation [reduced inhibitor of nuclear factor κB kinase α/β (IKKα/β) and p65 phosphorylation] (Fig. 2, H and I). We also observed a marked decrease in p38 MAPK phosphorylation in TLR2- and TLR10-depleted cells in OIS (Fig. 2H). These results show that TLR2 signaling is a significant contributor to the activation of the SASP by controlling NF-κB and p38 MAPK signal transduction pathways. Together, these data indicate that TLR2 signaling is necessary for the induction of the SASP.

### TLR2 reinforces the cell cycle arrest program in OIS

**Fig. 3:**
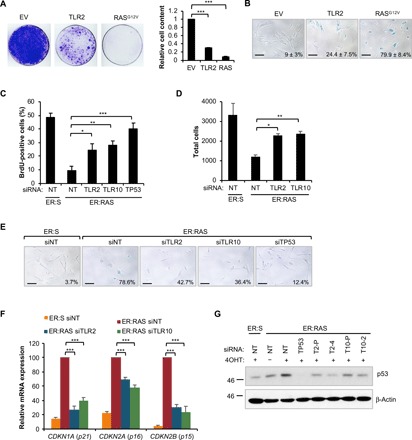
TLR2 reinforces the cell cycle arrest in OIS. (**A**) IMR90 cells infected with TLR2 or H-Ras^G12V^ expression vectors or empty vector (EV) control were seeded at low density and stained with crystal violet after 2 weeks. The staining was quantified to obtain relative cell content. Results are expressed as means ± SEM of three independent experiments. (**B**) SA-β-Gal staining was carried out on TLR2- and H-Ras^G12V^–expressing cells. Results are expressed as means (% positive cells) ± SEM of three independent experiments. (**C** to **G**) IMR90 ER:RAS cells were treated with 4OHT and repeatedly transfected with indicated siRNA and pooled nontarget siRNA control. siTP53 was used as a positive control. (C) After 5 days of treatment, a BrdU incorporation assay was conducted. Results are expressed as means ± SEM of three independent experiments. (D) Total DAPI-stained nuclei counted by high-content analysis at 8 days. Results are expressed as means ± SEM of three independent experiments. (E) After 10 days, SA-β-Gal activity assay was conducted. Scale bars, 100 μm. (F) qRT-PCR analysis of *CDKN1A*, *CDKN2A*, and *CDKN2B* transcripts. Results are expressed as means ± SEM of three independent experiments. (G) Western blot for p53 expression at 8 days. All statistical significance was calculated using one-way analysis of variance (ANOVA). ***P < 0.001, **P < 0.01, and *P < 0.05.

Activation of the SASP reinforces the cell cycle arrest program in senescence (*8*, *10*). Moreover, p38 MAPK is a significant regulator of OIS, controlling the activation of p53 and p16^INK4a^ tumor suppressor genes (*34*). Inhibition of p38 MAPK, but not of NF-κB, with chemical compounds bypassed the proliferative arrest and impaired p21^CIP1^ activation in IMR90 ER:RAS cells, showing a role for p38 MAPK in the reinforcement of the cell cycle arrest in OIS (fig. S4, A and B). Given the integral role TLR2 plays in the regulation of the SASP and p38 MAPK, we explored whether TLR2 also controls the cell cycle arrest in OIS. Overexpression of TLR2 induced cell cycle arrest and an increase in the number of senescence-associated β-galactosidase (SA-β-Gal)–positive cells (Fig. 3, A and B). Analysis of proliferation after ER:RAS activation showed that targeting TLR2 and TLR10 with siRNA strongly reduced the cell cycle arrest associated with OIS (Fig. 3C) and increased the long-term growth of ER:RAS cells (Fig. 3D and fig. S4, C and D). This effect correlated with a decrease in the number of cells positive for SA-β-Gal activity 10 days after ER:RAS activation (Fig. 3E). The control of proliferation by TLRs was specific for TLR2 and TLR10, as knockdown of the other eight members of the human TLR family did not show bypass of the cell cycle arrest program (fig. S4E). Suppression of TLR2 and TLR10 resulted in a decrease in p21^CIP1^, p16^INK4a^, and p15^INK4b^ mRNA expression (Fig. 3F) and a reduction of p53 protein levels (Fig. 3G). In addition, TLR10 knockdown induced escape of cell cycle arrest in cells subjected to conditioned media from OIS cells, suggesting a role in cell cycle arrest during paracrine senescence (fig. S4F). Together, these results indicate that TLR2 contributes to the cell cycle arrest program induced during OIS.

### TLR2 controls the expression of acute-phase serum amyloids A1 and A2, which are components of the SASP

**Fig. 4:**
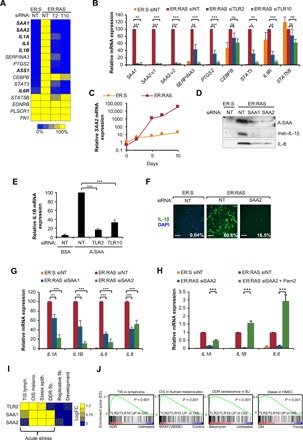
A-SAA signaling through TLR2 controls the SASP. (**A**) Heat map showing the relative fold change of acute-phase response transcripts of samples from the acute-phase response gene set from the mRNA transcriptomes. Transcriptome analysis (AmpliSeq) was performed in mRNA from IMR90 ER:RAS cells transfected with pooled siRNA for TLR2 and TLR10 and nontarget pool as a control. Genes with significant changes between nontarget siRNA control and both TLR2 and TLR10 knockdown are in bold characters. Adjusted *P* values were calculated using Benjamini and Hochberg false discovery rate of three independent experiments. Bold genes represent adjusted *P* < 0.05. (**B**) qRT-PCR validation of acute-phase response targets from samples obtained similarly to (A). Results are expressed as means ± SEM of three independent experiments. Statistical significance was calculated using one-way ANOVA and Dunnett’s multiple comparisons tests. ***P < 0.001, **P < 0.01, and *P < 0.05. (**C**) qRT-PCR analysis of A-SAA expression in IMR90 ER:RAS and ER:STOP cells with up to 10 days of 4OHT treatment. (**D**) IMR90 ER:RAS cells were treated with 4OHT and repeatedly transfected with pooled siRNA targeting SAA1 and SAA2 and nontarget siRNA as control for 8 days. Western blot of the conditioned medium for indicated antibodies. (**E**) IMR90 cells transfected with pooled siRNA for TLR2 and TLR10 were treated with A-SAA (10 μg/ml) for 3 hours, and qRT-PCR was performed to measure *IL1*β expression. Results are expressed as means ± SEM of three independent experiments. (**F**) Immunofluorescence staining and quantification of IL-1β expression by high-content analysis. Scale bars, 250 μm. (**G**) qRT-PCR for *IL1*α, *IL1*β, *IL6*, and *IL8* expression. Results are expressed as means ± SEM of three independent experiments. (**H**) IMR90 ER:RAS cells were transfected with siSAA2 and treated with 1 μm Pam2CSK4 for 5 days. qRT-PCR of *IL1*α, *IL1*β, and *IL6* expression. (**I**) Heat map showing TLR2 SAA1 and SAA2 expression in available transcriptomic data from adriamycin (ADR) mediated therapy-induced senescence (TIS) lymphoma cells (GSE31099), OIS mediated by mutant BRAF in human melanocytes (OIS) (GSE46801), stasis in human mammary epithelial cells (HMEC) (GSE16058), DNA damage-induced senescence in BJ cells (DDR) (GSE13330), replicative senescence in BJ cells (replicative) (GSE13330), and developmental senescence in the mesonephros (developmental) (GSE49108). (**J**) GSEA plots for the 267 genes regulated coregulated by TLR2 and TLR10 in OIS (fig. S2H) in the transcriptomes from (I). All statistical significance was calculated using one-way ANOVA. ***P < 0.001, **P < 0.01, and *P < 0.05.

GSEA of the transcriptome of TLR2- and TLR10-targeted cells revealed “acute-phase response” and “positive regulation of acute inflammatory response” as the top gene ontology terms (highest enrichment score and significance) for both conditions (fig. S5A). The acute-phase response is a clinical indication of an inflammatory event such as infection, trauma, or neoplasia and is characterized by the production and secretion of acute-phase proteins, including C-reactive protein and acute-phase serum amyloids by the liver (*35*). The two genes most strongly down-regulated by TLR2 and TLR10 knockdown were acute-phase serum amyloids A1 and A2 (*SAA1* and *SAA2*; A-SAAs hereinafter) (Fig. 4A). Other genes annotated as acute-phase response, such as *IL-1*α, *IL-1*β, *IL-6*, *SERPINA3*, *ASS1*, and *PTGS2*, also showed strong regulation by TLR2 and TLR10 in OIS (Fig. 4A). Further mRNA analysis by quantitative reverse transcription polymerase chain reaction (qRT-PCR) of SAA1, SAA2, SERPINA3, PTGS2, STAT3, and IL-6R expression confirmed these results (Fig. 4B), indicating that the expression of A-SAAs is highly induced during OIS and is dependent on TLR2 and TLR10. A time-course analysis of OIS revealed that A-SAA expression is induced 5 days after the activation of oncogenic RAS with 4OHT, which overlaps with TLR2 induction (Figs. 1C and 4C, and fig. S5B). Treatment of IMR90 cells with Pam2CSK4 (a synthetic agonist for TLR2) induced A-SAA expression, which was increased by one order of magnitude by ectopic overexpression of TLR2 (fig. S5C), suggesting a role for TLR2 sensing in the induction of A-SAAs in OIS. Western blot of the conditioned medium from IMR90 ER:RAS cells showed an accumulation of A-SAAs, which was reduced following siRNA knockdown of SAA1 and SAA2 (Fig. 4D and fig. S5D), suggesting that A-SAAs are components of the SASP. Similar to TLR2, A-SAA induction was associated to genotoxic stress. While induction of senescence with a genotoxic agent such as etoposide induced the expression of A-SAAs, direct induction of p53 activity with Nutlin 3a or by CDK4 inhibition with palbociclib failed to activate A-SAA expression (fig. S5E). We then studied the induction of A-SAAs in senescence in vivo. mRNA expression of *saa2* was induced in the lung of *nfkb1* knockout mice in parallel with the senescence markers cdkn1a (p21) and cdkn2a (p16) (fig. S5F). Moreover, increased expression of Saa1 was detected in early, low-proliferative PanINs in the KC mouse (fig. S5G). In summary, these results show that A-SAAs are components of the SASP regulated by TLR2.

### A-SAAs are potent senescence-associated DAMPs that control the SASP through TLR2 signaling

Previous reports have shown that A-SAAs have cytokine-like activity by interacting with TLR2 (*36*). Thus, we decided to explore the role of A-SAAs regulating the SASP in OIS. Recombinant A-SAA (rA-SAA) induced IL-1β mRNA expression with a similar effect to that achieved with an equimolar dose of the synthetic TLR2 agonist Pam2CSK4 (fig. S6A). Also, TLR2 overexpression in IMR90 cells enhanced rA-SAA–dependent induction of the SASP (fig. S6, B and C). Interference of TLR2 function with neutralizing antibodies (fig. S6D) or with siRNA against TLR2 and TLR10 (Fig. 4E and fig. S6E) also inhibited the rA-SAA–mediated induction of SASP components and SAA2. These results suggest that A-SAAs directly activate TLR2, resulting in priming of the inflammasome and SASP induction. To further confirm this, we decided to target the expression of A-SAAs during OIS with siRNA, showing that the induction of IL-1β and the SASP (Fig. 4, F and G) and the accumulation of mature IL-1β and IL-8 in conditioned media (Fig. 4D) were impaired when A-SAA was targeted. Moreover, treatment of IMR90 ER:RAS cells transfected with SAA2 siRNAs with the TLR2 agonist Pam2CSK4 rescued the induction of the SASP (Fig. 4H), confirming that TLR2 requires the presence of A-SAAs or additional exogenous DAMPs for the activation of the SASP. Together, these data indicate that A-SAAs are the DAMPs that signal through TLR2 to prime the inflammasome and regulate the SASP in OIS.

To assess the relative importance of TLR2- and A-SAA–mediated SASP regulation, we compared the effect of TLR2, TLR10, and SAA2 with other previously described regulators of the SASP, including the DNA damage response (DDR) pathway [ataxia-telangiectasia mutated (ATM)], the cyclic GMP-AMP synthase - stimulator of interferon genes (cGAS-STING) cytosolic DNA sensing pathway, the master SASP regulator IL-1 (IL-1R), the mechanistic target of rapamycin (mTOR) pathway, and the essential SASP transcription factors NF-κB (RELA) and C/EBPβ (*37*). Knockdown of the A-SAA–TLR2 pathway had a similarly negative effect on IL-1β induction to that following knockdown of the other known SASP regulators (fig. S6, F and G). In addition, knocking down the other A-SAA Toll-like receptor TLR4 did not decrease IL-1β, suggesting a specific role for A-SAA–TLR2 signaling in OIS (fig. S6G).

Last, we wanted to assess the existence of this regulatory pathway in additional cell types and senescence triggers. We first explored the expression of TLR2, SAA1, and SAA2 in available transcriptomic datasets of senescence (Fig. 4H). This analysis revealed that the A-SAA–TLR2 pathway was induced in senescence activated by acute stresses, such as therapy-induced senescence in lymphoma cells (*38*), BRAF^V600E^-induced senescence in human melanocytes (*39*), stasis in primary human mammary epithelial cells (*40*), and DDR-induced senescence in human dermal fibroblasts (BJ) (*41*), while it was not induced in replicative senescence (*41*) and programmed developmental senescence in the murine mesonephros (Fig. 4H) (*42*). Notably, the gene set composed of the 267 genes coregulated by TLR2 and TLR10 in OIS (Fig. 2D) was significantly enriched (*P* < 0.01) only in those transcriptomes with activation of A-SAA–TLR2 expression (Fig. 4I), while this correlation was not found with the transcriptome of replicative and developmental senescence (fig. S5H), suggesting a role for the A-SAA and TLR2 pair in senescence induced by several acute stresses in distinct cell types. Together, these results indicate that A-SAAs and TLR2 establish a master innate immune pathway with a key role in the control of the transcriptome in senescence.

### The cGAS-STING cytosolic DNA sensing pathway controls the induction of TLR2 and A-SAAs in OIS

**Fig. 5:**
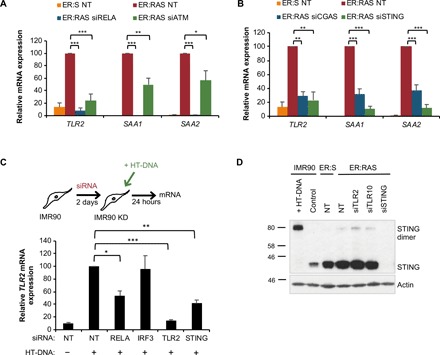
A-SAA and TLR2 expression is dependent on STING activation. (**A** and **B**) IMR90 ER:RAS cells were treated with 4OHT and repeatedly transfected with indicated pooled siRNA and nontarget siRNA as control for 8 days. *TLR2*, *SAA1*, and *SAA2* transcripts were measured by qRT-PCR. Results are expressed as means ± SEM of three independent experiments. (**C**) IMR90 cells were transfected with siRNA targeting RELA, IRF3, TLR2, and STING for 2 days, followed by transfection with 2.5 μg of herrings-testes DNA (HT-DNA) for 24 hours. *TLR2* transcripts were measured by qRT-PCR. Results are expressed as means ± SEM of three independent experiments. (**D**) Western blot for STING dimerization. HT-DNA transfection of IMR90 cells were used as positive control for STING dimerization. IMR90 ER:RAS cells were transfected with siRNA targeting TLR2, TLR10, and STING for 8 days with 4OHT. All statistical significance was calculated using a one-way ANOVA. ***P < 0.001, **P < 0.01, and *P < 0.05.

Experiments targeting RELA and ATM revealed that the activation of TLR2 and A-SAAs is dependent on NF-κB and the DDR in OIS (Fig. 5A). Recently, it has been shown that, in senescence, NF-κB is activated by the cGAS-STING cytosolic DNA sensing pathway by the accumulation of cytoplasmic chromatin fragments released from the damaged nucleus in response to genotoxic stress (*43*). Thus, we speculated that this pathway could be responsible for the induction of TLR2 and A-SAAs in OIS. Inactivation of cGAS and STING with siRNA in OIS strongly impaired the transcriptional activation of TLR2, SAA1, and SAA2 mRNA expression (Fig. 5B), indicating a role for the DNA sensing pathway in the regulation of TLR2 and A-SAAs. Moreover, direct activation of cGAS-STING by transfecting double-stranded DNA (dsDNA) into IMR90 cells induced the formation of STING homodimers, which is a hallmark of its activation, and induced TLR2 expression, which was reduced by STING knockdown (Fig. 5, C and D). STING signals through the NF-κB and the interferon response pathways to transcriptionally activate its target genes. To elucidate how the cGAS-STING pathway induces the expression of TLR2 and A-SAAs, we knocked down RELA and IRF3 in cells transfected with dsDNA, showing that the inactivation of the NF-κB pathway with siRNA against RELA, but not IRF3, impaired TLR2 and A-SAA induction in IMR90 cells (Fig. 5C and fig. S6, I and J). Moreover, in contrast to RELA knockdown (Fig. 5A), IRF3 targeting did not impair TLR2 induction in OIS (fig. S6K), suggesting a specific role for the NF-κB pathway downstream of STING in TLR2 induction in OIS. Last, knockdown of TLR2 and TLR10 did not reduce the formation of STING homodimers in OIS (Fig. 5D), suggesting that TLR2 signaling is the downstream of the cGAS-STING pathway. Together, these results indicate that A-SAAs and TLR2 are downstream of the cGAS-STING cytosolic DNA sensing pathway in OIS.

### TLR2 regulates the SASP and OIS in vivo

**Fig. 6:**
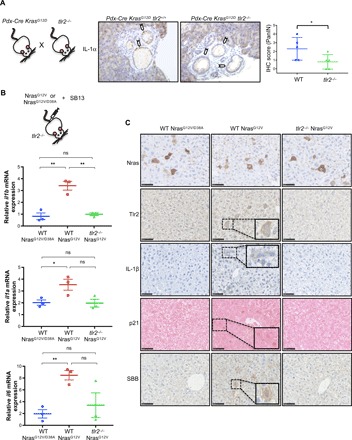
*tlr2* is necessary for SASP activation in vivo. (**A**) Representative IHC staining and IHC score quantification of IL-1α in PanIN generated in *tlr2^+/+^* or *tlr2^−/−^ Pdx-Cre Kras^G12D^* mice. Scatter plot represents the value for individual animals (dots), and the horizontal line represents group means (*n* = 5) ± SEM. Statistical significance was calculated using one-tailed Student’s *t* test. *P < 0.05 (**B**) qRT-PCR results for SASP factors IL-1β, IL-1α, and IL-6 from liver samples from WT and *tlr2^−/−^* mice 6 days after receiving hydrodynamic delivery of Nras^G12V/D38A^ negative control or oncogenic Nras^G12V^ transposon as indicated. Scatter plot represents the value for individual animals (dots), and the horizontal line represents group means (*n* = 3) ± SEM. Statistical significance was calculated using two-tailed students *t* test. *P < 0.05 and **P < 0.01. (**C**) Representative IHC staining for Nras, Tlr2, IL-1β, p21, and Biotin-SBB in corresponding liver sections from mice in (B). Scale bars, 50 μm.

The strong enrichment of the TLR2/TLR10 OIS-dependent gene set in the publically available transcriptome of Kras^G12D^-driven PanINs in KC mice (*44*) suggested a role for TLR2 in this in vivo model (fig. S7A). Therefore, we decided to test the effect of knocking out *tlr2* (*tlr2^−/−^*) on SASP activation in KC mice. We observed a reduction in IL-1α staining in PanIN epithelial cells of *tlr2^−/−^* mice compared to wild-type (WT) mice (Fig. 6A), indicating a causal role for TLR2 in the activation of the SASP in PanIN. We also assessed the effect on SASP expression after hydrodynamic delivery of Nras^G12V^ transposons into hepatocytes of *tlr2^−/−^* mice (Fig. 6, B and C). The transduction of oncogenic Nras^G12V^, but not of the inactive Nras^G12V/D38A^, induced IL-1β, IL-1α, and IL-6 mRNA expression (Fig. 6B) and the accumulation of IL-1β– and Tlr2-positive hepatocytes (Fig. 6C) in WT mice. In contrast, Nras^G12V^ failed to induce IL-1β– and Tlr2-positive hepatocytes and IL-1β mRNA expression in the liver of *tlr2^−/−^* mice (Fig. 6, B and C). Moreover, the induction of other SASP components, IL-1α and IL-6, was also impaired in *tlr2^−/−^* mice (Fig. 6B). Nras^G12V^-induced senescence, measured by the increase in p21 or biotinylated Sudan Black B (Biotin-SBB)–positive hepatocytes (Fig. 6C) and in SA-β-Gal activity in the whole liver (fig. S7B), was impaired in *tlr2^−/−^* mice. In summary, these results indicate that *tlr2* is necessary for the induction of the SASP and the activation of OIS in vivo.

## DISCUSSION

We describe here an essential innate immune signaling pathway in OIS established between TLR2 and A-SAAs that initiates the SASP and reinforce cellular senescence in vitro and in vivo (fig. S7C). We also identify new important SASP components, A-SAAs, which are the senescence-associated DAMPs sensed by TLR2 after oncogenic stress. Therefore, we are reporting that innate immune sensing is critical in senescence.

We propose that cellular senescence shares mechanistic features with the activation of innate immune cells and could be considered a program of the innate immune response by which somatic cells switch their regular role to acquire an immune function under certain conditions of stress and danger, for instance, upon oncogene activation. In OIS, super-enhancer elements regulated by Bromodomain-containing protein 4 (BRD4) adjacent to the SASP have been shown to regulate the immune surveillance of senescent cells (*45*). In the same study, it was demonstrated that TLR pathways in general, and TLR2 in particular, were associated with activation of typical enhancers during OIS, suggesting that enhancer remodeling might shape the switch that activates this immune sensing program during senescence. In addition, cytosolic DNA sensing by the innate immune sensor cGAS has also been identified as an essential step in the activation of the SASP (*43*, *46*). In our study, we have shown that the induction of TLR2 and A-SAAs depends on the activation of cGAS and STING in senescence, and they appear to function downstream of STING to regulate the SASP and NF-κB (fig. S7C). Future research will be necessary to understand further the cross-talk between both pathways in senescence. For example, Dou *et al*. proposed that p38 MAPK signaling impairs the activation of the interferon response arm of the cGAS-STING pathway, prioritizing the NF-κB response (*43*). In agreement, our data suggests that TLR2 and A-SAA expression is regulated through the NF-κB, but not the IRF3, pathway downstream of cGAS-STING. We observed that TLR2 is essential for both the steady-state activation of p38 MAPK and NF-κB during OIS. Therefore, it is tempting to speculate that TLR2 may interact with the cGAS-STING pathway in senescence by actively impairing the activation of interferon response genes. As a result of these net interactions between innate immune receptors, TLR2 signaling is critical for the SASP and the cell cycle arrest and coincides with the role of both p38 MAPK and NF-κB signal transduction pathways in OIS (*8*, *10*, *22*, *34*).

In our experiments, we observed that TLR10 regulates a larger number of genes than TLR2. In contrast to the TLR2/10 coregulated genes, TLR10-specific genes were not directly related to inflammation. TLR10 is a largely under-characterized gene with undefined function, and in mice, it is a pseudogene inactivated by serial retrotransposition (*32*). Our study suggests a potential specific role for TLR10 in cellular senescence in human cells, with some possible TLR2-independent functions. However, further research will be necessary to clarify whether this subject is real or is the result of the higher efficiency of the siRNA reagent or the relative abundance between TLR2 and TLR10.

Besides revealing a role for TLR2 in SASP induction and cell cycle regulation, we identified the DAMP that activates TLR2 in OIS. Acute-phase proteins SAA1 and SAA2 act to prime the TLR2-mediated inflammasome, and in turn, their full induction depends on TLR2 function. Hence, they establish a foundational feedback loop that controls the SASP (fig. S7C). A-SAAs are systemically produced in the liver and released into the bloodstream during an acute inflammatory response (*35*). Our identification of these molecules as mediators of senescence suggests that systemic elevation of A-SAAs might have an impact on the accumulation of senescent cells and the activation of their proinflammatory program at the organismal level. We found activation of TLR2 expression in parallel to A-SAAs in models of OIS in mice, in inflammation-induced senescence, in aging, and in different in vitro systems of senescence. Also, we have shown that TLR2 controls the activation of the SASP and OIS in vivo. Moreover, we have observed a dose-dependent effect for TLR2 in A-SAA sensing and a role for TLR2 in SASP activation during paracrine senescence. Together, these data suggest that systemic A-SAA elevation during acute inflammation could affect cells expressing TLR2, thereby promoting aging and other pathological roles of senescence. Further investigation may reveal additional physiological circumstances under which senescence is induced or reinforced by the interaction of TLR2 with A-SAAs or indeed with other endogenous DAMPs or exogenous PAMPs from the microbiome. These circumstances could have implications for organismal well-being, in particular, the development of aging and cancer.

Last, in recent years, several strategies have been implemented to eliminate senescent cells or to modulate the activation of the SASP in anti-aging and cancer therapies (senotherapies) (*47*–*49*). For example, genetic targeting for the elimination of senescent cells can delay organismal aging and aging-associated disorders (*50*, *51*). Furthermore, the pharmacological suppression of the SASP has been shown to improve homoeostasis in tissue damage and aging (*49*). However, most of these manipulations are directed to essential homeostatic regulators such as mTOR or crucial proinflammatory mediators such as IL-1 signaling. Here, we propose the alternative of manipulating A-SAA–TLR2 as a new rationale for senotherapies aiming to manipulate nonessential and senescence-specific signaling pathways.

## MATERIALS AND METHODS

### Cell culture

Human embryonic kidney (HEK) 293T and IMR90 female human fetal lung fibroblast cells were obtained from American Type Culture Collection. All cells were maintained in Dulbecco’s modified Eagle’s medium (DMEM) (Thermo Fisher Scientific) supplemented with 10% fetal bovine serum (FBS) (Thermo Fisher Scientific) and 1% antibiotic-antimycotic solution (Thermo Fisher Scientific). IMR90 cells were kept at 5% CO_2_ and ambient O_2_ at 37°C. All cell lines were regularly tested for mycoplasma contamination using the MycoAlert Mycoplasma Detection Kit (Lonza). Cell counting and viability were performed using the Muse Count and Viability Assay Kit in a Muse Cell Analyzer (Merck Millipore).

### Experiments with mice

All work was compiled with the U.K. Home Office guiding principles for the care and use of laboratory animals. Mice carrying a conditional *Pdx1-Cre Kras^G12D/+^* allele were used and have been described previously (*26*). Aging experiments were carried out on male WT C57BL/6 mice or male *nfkb1^−/−^* mice on a pure C57BL/6 background at 6.5, 9.5, and 24 months of age. For hydrodynamic tail vein injection experiments, *tlr2^−/−^* mice on a C57BL/6 background were purchased from the Jackson Laboratory (JAX). Male and female *tlr2^−/−^* mice and WT siblings, aged between 8 to 12 weeks, were included in the study. Plasmids for hydrodynamic injection were prepared using the Qiagen Plasmid Maxi Kit (Qiagen, Germany), as per the manufacturer’s instructions. Animals received 6 μg of a sleeping beauty transposase–encoding plasmid (CMV-SB 13 transposase), and 20 μg of NRas^G12V^/green fluorescent protein (GFP) encoding plasmid (pT3-NRas^G12V^-IRES-GFP) was diluted in physiological saline to 10% of the animal’s body weight (approximately 2 ml) and delivered via the lateral tail vein within 10 s. Plasmid encoding an NRas^G12V^ effector loop mutant (pT3-NRas^G12V/D38A^-IRES-GFP), incapable of downstream NRas signaling, was used as a control. Mice were culled after 6 days, and liver tissue was harvested.

### Chemical compounds and neutralizing antibodies

OIS was induced by treating IMR90 ER:RAS cells with 100 nM 4OHT (Sigma-Aldrich). IMR90 ER:STOP was used as a negative control. To induce senescence, IMR90 cells were treated with 100 μM etoposide for 48 hours, followed by 5 days in normal culture media, or with 10 μM palbociclib or 10 μM Nutlin 3a for 7 days. TLR2 was blocked by incubating cells with anti-TLR2 (10 μg/ml) (MAB2616, R&D system) or immunoglobulin G2B isotype control. Chemical inhibitors used were 10 μM BAY-117082 (Calbiochem), 10 μM SB202190 (Calbiochem), and OxPAPC (100 μg/ml) (Invivogen). TLR2 in IMR90 cells was primed with recombinant human Apo-SAA (10 μg/ml; PeproTech, catalog no. 300-30) or Pam2SK4 (1 μg/ml; Tocris, catalog no. 4637).

### Conditioned medium for paracrine senescence transmission

For the production of conditional medium used in paracrine senescence, IMR90 ER:STOP or ER:RAS cells were cultured with 100 nM 4OHT in DMEM supplemented with 10% FBS for 4 days, followed by DMEM supplemented with 1% FBS and 4OHT for an additional 4 days. The resulting conditioned medium was filtered with 0.2-μm syringe filters (Millipore) and reconstituted with a solution of DMEM supplemented with 40% FBS at a ratio of 3:1.

### Construction of plasmids

TLR2 complementary DNA (cDNA) was amplified from the pcDNA3-TLR2-YFP plasmid (Addgene, 13016) using primers flanked with Xho I sites. Amplified genes were cloned into the MSCV-puro vector. pLN-ER:RAS, LSXN-ER:Stop, MSCV-Ras^G12V^, CMV-VSVG, and pNGVL-Gag-Pol vectors were described elsewhere (*15*).

### Retroviral production and infection

For retroviral production, retroviral vectors were cotransfected with VSV-G envelope plasmid and Gag-Pol helper vector using polyethylenimine linear (molecular weight, 25.000; Polysciences) into HEK293T cells. Viral supernatant was collected from the HEK293T cells 2 days after transfection and passed through a 0.45-μm syringe filter (Merck Millipore) to eliminate cells. The viral supernatant was supplemented with hexadimethrine bromide (4 μg/ml; polybrene) (Sigma-Aldrich) and used to incubate IMR90 cells, with subsequent viral supernatant collection and treatment of IMR90 cells every 3 hours. After three rounds of infection, the medium was changed to fresh DMEM with 10% FBS, and cells were allowed to grow for 2 to 3 days before selection with puromycin (1 μg/ml) (Invivogen) for another 7 days before seeding for indicated experiments.

### Immunohistochemistry

Formalin-fixed, paraffin-embedded sections were dewaxed, rehydrated through graded ethanol solutions (100, 90, and 70%), and washed in distilled H_2_O. Endogenous peroxidase activity was blocked by immersing sections H_2_O_2_ (H1009, Sigma). To retrieve antigens, sections were boiled in 0.01 M citrate (pH 6.0), except for Tlr2 staining where antigens were retrieved using proteinase K (20 μg/ml). Sections were incubated with the primary antibody overnight at 4°C (antibody information is provided in table S3). For the *Pdx1-Cre Kras^G12D/+^* mice experiment, the EnVision+ Dual Link System-HRP (DAB+) Kit (K4065, Dako) was used for Ki67 staining. The total number of Ki67-positive cells per PanIN and the total cells per PanIN were counted, and thus, the percentage of Ki67-positive cells per PanIN was calculated. The mean score for each mouse was calculated, and these scores were plotted on a box plot. Consecutive sections were stained with antibodies against Tlr2 and Saa1. For the *nfkb^−/−^* mice experiment, biotinylated secondary antibody was added and detected using the rabbit peroxidase ABC Kit (PK-4001, Vector Laboratories), according to the manufacturer’s instructions. Substrate was developed using the NovaRED Kit (SK-4800, Vector Laboratories). Staining was analyzed with a Nikon Eclipse E800 microscope, and images were captured with a Leica DFC420 camera using the LAS software (Leica). Ten to 15 random images were captured per section, and the percentage of positively stained cells determined from total number of cells before an average per mouse was calculated. For the hydrodynamic tail vein injection experiment, biotinylated secondary antibodies were added and detected using the R.T.U Vectastain Kit (PK-7100, Vector). Substrate was developed using the DAB substrate Kit (ab64238, Abcam). Staining was analyzed with a Hamamatsu NanoZoomer XR microscope, and images were captured using the NDP scan v.3.1 software (Hamamatsu).

### Whole-mount SA-β-Gal staining

Snap-frozen liver samples were thawed before fixing in 2% formaldehyde/0.2% glutaraldehyde solution for 30 min at room temperature. Samples were subsequently washed with phosphate-buffered saline (PBS) and incubated at 37°C overnight in the dark in β-Gal staining solution [5 mM K_3_FE (CN)_6_ and 5 mM K_4_Fe (CN) _6_*3H_2_O in PBS, X-Gal solution (1 mg/ml), 150 mM NaCl, and 2 mM MgCl_2_ at pH 6 in citric acid/phosphate buffer]. Samples were then washed, dehydrated, and imaged.

### Biotin-SBB detection in paraffin-embedded sections

For Biotin-SBB IHC, paraffin-embedded sections were dewaxed, rehydrated, and blocked as per other IHC samples. Samples were immersed in additional graded ethanol washes before incubation with a Biotin-SBB analog (SenTraGor), which selectively binds to lipofuscin, for 8 min at room temperature. Samples were washed and then incubated with primary antibiotin antibody (see table S3) overnight at 4°C. The samples were then incubated with a horseradish peroxidase–conjugated secondary antibody for 1 hour at room temperature, and then, substrate was developed using the DAB substrate Kit (ab64238, Abcam). Staining was analyzed with a Hamamatsu NanoZoomer XR microscope, and images were captured using the NDP scan v 3.1 software (Hamamatsu). The antibodies used are in table S3.

### Herrings-testes DNA transfection

MR90 cells were seeded into six-well tissue culture plates, incubated overnight, and transfected with 2500 ng of herrings-testes DNA using Lipofectamine 2000, following the manufacturer’s protocol. Samples collected for analysis 24 hours after transfection.

### siRNA transfection

Reverse siRNA transfection was carried out using 30 nM siRNA (Dharmacon, GE Healthcare). siRNA sequences are provided in table S2. DharmaFECT 1 (Dharmacon, GE Healthcare) transfection reagent was diluted in DMEM and added to the siRNA-containing wells. This complex was allowed to form while cells were trypsinized and prepared for plating. IMR90 ER:RAS or ER:STOP cells (2000 cells per well in a 96-well plate; 66,000 cells per well in a six-well plate) were plated into the siRNA-containing wells with 100 nM 4OHT for the activation of ER:RAS. Because of the transient nature of siRNA to maintain the knockdown for up to 8 days, the cells were forward-transfected with medium containing the same proportions of siRNA, transfection reagent, and 4OHT on days 3 and 5. A list of the siRNA reagents is provided in table S2.

### Total RNA preparation and quantitative reverse transcription polymerase chain reaction (qRT-PCR)

RNA was extracted from IMR90 cells using the RNeasy Plus Kit (Qiagen), following the manufacturer’s instructions. For mRNA expression in murine livers, snap-frozen specimens were homogenized with trizole, and RNA was extracted using the Qiagen RNeasy plus mini kit (Qiagen, Germany), according to the manufacturer’s instructions. cDNA was synthesized using qScript cDNA SuperMix (Quanta Biosciences), following the manufacturer’s instructions, from 1 μg of RNA in a 40-μl reaction. qRT-PCR was performed using 1 μl of cDNA as a template per reaction well and SYBR Select Master Mix (Life Technologies) using 200 nM of forward and reverse primers in 20 μl. Samples were run in triplicate on a StepOnePlus Cycler (Thermo Fisher Scientific).

For the nfkb^−/−^ aging experiment, RNA was extracted from solubilized lung tissue using the RNeasy Mini Kit (74106, Qiagen), according to the manufacturer’s instructions. cDNA was generated using the Omniscript RT Kit (205110, Qiagen) as per the user manual. qRT-PCR was performed using 4 μl of cDNA as a template per reaction well using a Power SYBR Green (4367659, Invitrogen) PCR Master Mix and 100 nM of forward and reverse primers to form a final reaction volume of 10 μl. Samples were run in triplicate in a C1000TM Thermal Cycler, CFX96TM Real-Time System (Bio-Rad), and Bio-Rad CXF manager software.

mRNA expression analysis was carried out using the change in Ct method and normalized to levels of the housekeeping gene actin or ribosomal protein 18*S* (nfkb^−/−^ experiment only) to obtain relative mRNA expression. All primers used are listed in the table S1.

### Immunofluorescence and high-content microscopy

All immunofluorescence staining and imaging was performed on the ImageXpress high-content analysis microscope (Molecular Devices), as previously described (*52*). All steps were carried out at room temperature. Following the indicated treatment, cells were fixed with 4% paraformaldehyde for 1 hour and permeabilized with 0.2% Triton X-100 for 10 min. After three washes with PBS, the cells were blocked with 0.2% fish-skin gelatin/bovine serum albumin (BSA)/PBS for 1 hour. Primary antibodies were diluted in blocking solution as indicated and incubated for 30 min, followed by three washes in PBS. Appropriately conjugated Alexa Fluor secondary antibodies were diluted 1:1000 in blocking solution and incubated on the cells for 40 min, followed by an additional three washes in PBS. Last, 4′,6-diamidino-2-phenylindole (1 μg/ml) was added to the cells for 20 min, after which the plates underwent a final round of washes before imaging on the ImageXpress microscope. Quantification of immunofluorescence images was conducted using the MetaXpress software (Molecular Devices), as previously described (*52*).

### Western blot analysis

Whole-cell lysates were prepared by lysing the cells with an appropriate volume of radioimmunoprecipitation assay buffer [10 mM tris (pH 7.4), 100 mM NaCl, 1 mM EDTA, 1 mM EGTA, 0.1% SDS, 1% Triton X-100, 1 mM β-mercaptoethanol, 0.5% sodium deoxycholate, 10% glycerol, phosphatase inhibitor cocktail III, protease inhibitor cocktail V, and EDTA free]. Lysates were incubated on ice for 15 min before centrifuging at 14,000 rpm for 20 min to clear lysates. Supernatants were collected into a clean tube and quantified using the Bradford assay. Samples were prepared for loading by mixing with Laemmli sample buffer and boiled at 95°C for 5 min. Protein samples were resolved by polyacrylamide gel electrophoresis and transferred onto nitrocellulose membrane using the iBlot Dry Blotting System (Thermo Fisher Scientific). Membranes were blocked for 1 hour in 5% milk/tris-buffered saline–1% Tween (TBST). Indicated primary antibodies were diluted in 5% milk/TBST or 5% BSA/TBST and incubated at 4°C with gentle agitation overnight. Blots were washed for a minimum of three times in TBST for more than 30 min. Secondary antibodies were prepared in 5% milk/TBST, and membranes were incubated at room temperature for 1 hour. Blots were washed as before, and then, enhanced chemical luminescence (Amersham) detection reagent was applied for detection. Antibody information is provided in table S3.

### Cell proliferation assays

The 5-bromo-2′-deoxyuridine (BrdU) incorporation assay was used to measure the number of cells actively replicating DNA. Cells were treated as indicated in 96-well plates and incubated with 10 μM BrdU (Sigma) for 16 to 18 hours before fixation and immunostained as described using anti-BrdU antibody with deoxyribonuclease (0.5 U/μl) (Sigma) in 1 mM MgCl containing PBS.

To visualize and quantify long-term growth, cells were plated at low density (50,000 cells/10-cm plate) and maintained for 10 to 15 days. The cells were fixed using 1% glutaraldehyde (Sigma) for 1 hour and dried overnight before staining with 0.15% crystal violet solution for 2 hours. The plates were then washed, dried, and scanned for documentation. For quantification, crystal violet was extracted from stained plates with 1% acetic acid and quantified by absorbance read at 595 nm.

### SA-β-Gal assay

SA-β-Gal staining solution was prepared using 20× KC [100 mM K_3_FE (CN)_6_ and 100 mM K_4_Fe (CN) _6_*3H_2_O in PBS], 20× X-Gal solution (Thermo Fisher Scientific) diluted to 1× in PBS/1 mM MgCl_2_ at pH 5.5 to 6. The cells were treated as indicated and fixed in 0.5% glutaraldehyde (Sigma) for 10 min at room temperature. Cells were washed twice in PBS/1 mM MgCl_2_ (pH 5.5 to 6) before incubation in 2 ml of staining solution for 24 hours at 37°C.

### AmpliSeq transcriptome profiling

RNA samples were assessed for quality on the Agilent Bioanalyzer with the RNA Nano Chip, providing an RNA Integrity Number. Samples were quantified using the Qubit 2.0 fluorometer and the Qubit RNA Broad Range assay. Ten nanograms of RNA was reverse-transcribed to make cDNA, and then, target genes were amplified for 12 cycles of PCR using the Ion AmpliSeq Human Gene Expression Core Panel. This panel contains 20,802 amplicons (41,604 primers) of approximately 150 bases in length in a single pool. Ion Torrent sequencing adapters and barcodes were ligated to the amplicons, and adapter-ligated libraries were purified using AMPure XP beads. Libraries were quantified by qPCR and diluted to 100 pM. Templates were prepared using the Ion PI Hi-Q OT2 200 Kit and sequenced using the Ion PI Hi-Q Sequencing 200 Kit. The Ion Proton platform was used to analyze the data. Analysis of gene expression was performed using the software package Babelomics 5.0 (*53*, *54*). Statistical analysis of significance was performed using Benjamini and Hochberg false discovery rate multiple test. GSEA was performed using GSEA 3.0 software from the Broad Institute (www.gsea-msigdb.org) (*55*). Gene ontology analysis was performed using DAVID functional annotation web resource (https://david-d.ncifcrf.gov).

### Determination of IL-1β in conditioned medium

Supernatant of medium from siRNA-treated cells was collected for the analysis of IL-1β content. The medium was combined with 6× Laemmli sample buffer, and IL-1β expression was determined by immunoblotting, as described. Conditional medium samples were also analyzed for IL-1β expression by enzyme-linked immunosorbent assay (ELISA) using the Human IL-1β ELISA Ready-Set-Go! Kit (Affymetrix eBioscience), following the manufacturer’s instructions.

### Quantification and statistical analysis

IHC images were quantified using ImageJ analysis software, and graphs and statistical analysis were carried out using GraphPad Prism version 7.0 (GraphPad Software, San Diego, CA).

## Supplementary Material

http://advances.sciencemag.org/cgi/content/full/5/6/eaaw0254/DC1

Download PDF

Data file S1

Data file S2

Data file S3

## SUPPLEMENTARY MATERIALS

Supplementary material for this article is available at http://advances.sciencemag.org/cgi/content/full/5/6/eaaw0254/DC1 Fig. S1. TLR2 expression is induced during OIS in vitro. Fig. S2. TLR2 expression is induced during OIS in vivo. Fig. S3. TLR2 and TLR10 regulate the SASP in OIS. Fig. S4. TLR2 reinforces the cell cycle arrest in OIS. Fig. S5. TLR2 and TLR10 regulate the activation of genes of the acute-phase response during OIS. Fig. S6. A-SAA signaling through TLR2 controls the SASP. Fig. S7. *tlr2* is necessary for OIS activation in vivo. Table S1. Primers used for qRT-PCR in this study. Table S2. siRNA sequences used in this study. Table S3. Antibodies used in this study. Data file S1. Transcriptome analysis of the effect of TLR2 and TLR10 siRNAs in OIS. Data file S2. Genes coregulated by TLR2 and TLR10 in OIS. Data file S3. Genes regulated only by TLR10 in OIS.
