# Supplementary material for: The innate immune sensor Toll-like receptor 2 controls the senescence-associated secretory phenotype
Source: Sci Adv. 2019 Jun 5;5(6):eaaw0254. doi: 10.1126/sciadv.aaw0254 (PMC6551188; doi:10.1126/sciadv.aaw0254)
Supplement: http://advances.sciencemag.org/cgi/content/full/5/6/eaaw0254/DC1 [file supp_5_6_eaaw0254__index.html]

Science Advances | Science Advances

## Supplementary Materials

**The PDF file includes:**

- Fig. S1. TLR2 expression is induced during OIS in vitro.
- Fig. S2. TLR2 expression is induced during OIS in vivo.
- Fig. S3. TLR2 and TLR10 regulate the SASP in OIS.
- Fig. S4. TLR2 reinforces the cell cycle arrest in OIS.
- Fig. S5. TLR2 and TLR10 regulate the activation of genes of the acute-phase response during OIS.
- Fig. S6. A-SAA signaling through TLR2 controls the SASP.
- Fig. S7. *tlr2* is necessary for OIS activation in vivo.
- Table S1. Primers used for qRT-PCR in this study.
- Table S2. siRNA sequences used in this study.
- Table S3. Antibodies used in this study.

Download PDF

**Other Supplementary Material for this manuscript includes the following:**

- Data file S1 (Microsoft Excel format). Transcriptome analysis of the effect of TLR2 and TLR10 siRNAs in OIS.
- Data file S2 (Microsoft Excel format). Genes coregulated by TLR2 and TLR10 in OIS.
- Data file S3 (Microsoft Excel format). Genes regulated only by TLR10 in OIS.

**Files in this Data Supplement:**

- Adobe PDF - aaw0254\_SM.pdf
