# Supplementary material for: The innate immune sensor Toll-like receptor 2 controls the senescence-associated secretory phenotype
Source: Sci Adv. 2019 Jun 5;5(6):eaaw0254. doi: 10.1126/sciadv.aaw0254 (PMC6551188; doi:10.1126/sciadv.aaw0254)
Supplement: Download PDF [file aaw0254_SM.pdf]

Science Advances
AAAS

advances.sciencemag.org/cgi/content/full/5/6/eaaw0254/DC1

Supplementary Materials for

The innate immune sensor Toll-like receptor 2 controls the senescence-associated secretory phenotype

Priya Hari, Fraser R. Millar, Nuria Tarrats, Jodie Birch, Andrea Quintanilla, Curtis J. Rink, Irene Fernández-Duran, Morwenna Muir, Andrew J. Finch, Valerie G. Brunton, João F. Passos, Jennifer P. Morton, Luke Boulter, Juan Carlos Acosta*

*Corresponding author. Email: juan-carlos.acosta@igmm.ed.ac.uk

Published 5 June 2019, *Sci. Adv.* **5**, eaaw0254 (2019)
DOI: 10.1126/sciadv.aaw0254

The PDF file includes:

Fig. S1. TLR2 expression is induced during OIS in vitro.
Fig. S2. TLR2 expression is induced during OIS in vivo.
Fig. S3. TLR2 and TLR10 regulate the SASP in OIS.
Fig. S4. TLR2 reinforces the cell cycle arrest in OIS.
Fig. S5. TLR2 and TLR10 regulate the activation of genes of the acute-phase response during OIS.
Fig. S6. A-SAA signaling through TLR2 controls the SASP.
Fig. S7. *tlr2* is necessary for OIS activation in vivo.
Table S1. Primers used for qRT-PCR in this study.
Table S2. siRNA sequences used in this study.
Table S3. Antibodies used in this study.

Other Supplementary Material for this manuscript includes the following:

(available at advances.sciencemag.org/cgi/content/full/5/6/eaaw0254/DC1)

Data file S1 (Microsoft Excel format). Transcriptome analysis of the effect of TLR2 and TLR10 siRNAs in OIS.
Data file S2 (Microsoft Excel format). Genes coregulated by TLR2 and TLR10 in OIS.
Data file S3 (Microsoft Excel format). Genes regulated only by TLR10 in OIS.

Figure S1

A

	Genes detected	Positive regulated	Negative regulated
IMR90 ER:RAS vs ER:S	10835	1523	2059

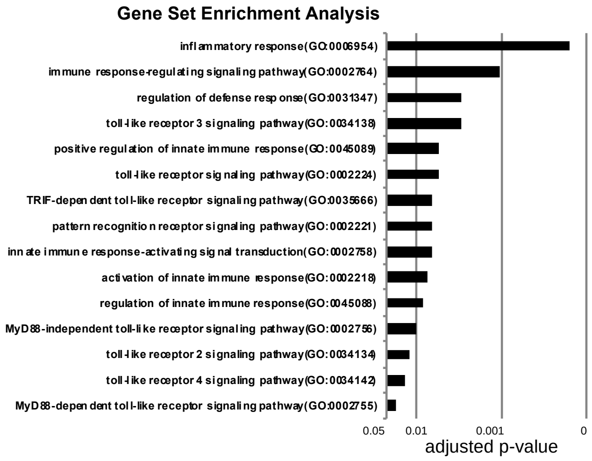

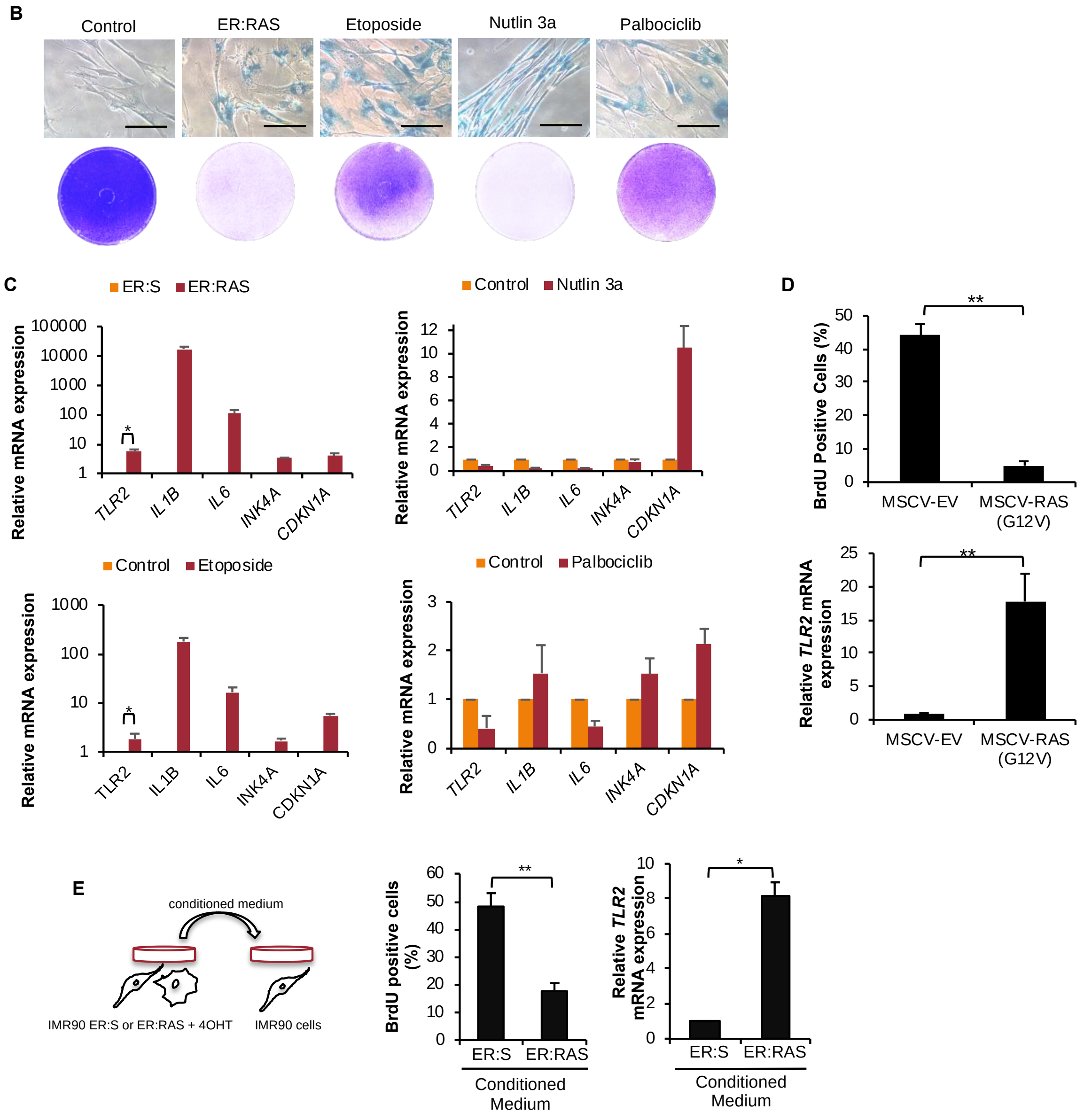

Fig. S1. TLR2 expression is induced during OIS in vitro. (**A**) Transcriptome analysis and Gene Set Enrichment Analysis was conducted to compare oncogene-induced senescent cells (IMR90 ER:RAS + 4OHT at 8 days) vs. control cells (IMR90 ER:Stop + 4OHT at 8 days) of 6 independent experiments. Adjusted p-value of significant gene sets related to the innate immune response and pattern recognition receptors are shown. (**B**) IMR90 cells were induced to senescence either ER:RAS activation, 100 μM Etoposide, 10 μM Nutlin 3a or 10 μM Palbociclib for 7 days. Representaive SA-β-Gal images at 20X magnification and crystal violet staining shown. (**C**) RT-qPCR of cells treated as in (C) for *TLR2, IL1B, IL6, INK4A* and *CDKN1A* expression. (**D**) BrdU incorporation assay and qRT-PCR of TLR2 mRNA expression of IMR90 cells infected with RAS^{G12V} vector and empty vector (EV) as control. (**E**) Conditioned medium (CM) generated by IMR90 ER:Stop and ER:RAS following 8 days of 4OHT treatment was transferred to proliferating IMR90 cells and cultured for 2 days. After a 16-hour pulse, BrdU incorporation was high content analysis. qRT-PCR of *TLR2* mRNA in IMR90 cells treated with CM from ER:RAS and ER:Stop cells was assessed. Results expressed as mean ± SEM of 3 independent experiments. Statistical significance was calculated using Students two-tailed *t*-test. $^{**}p < 0.01$, $^{*}p < 0.05$.

Figure S2

Hari et al.

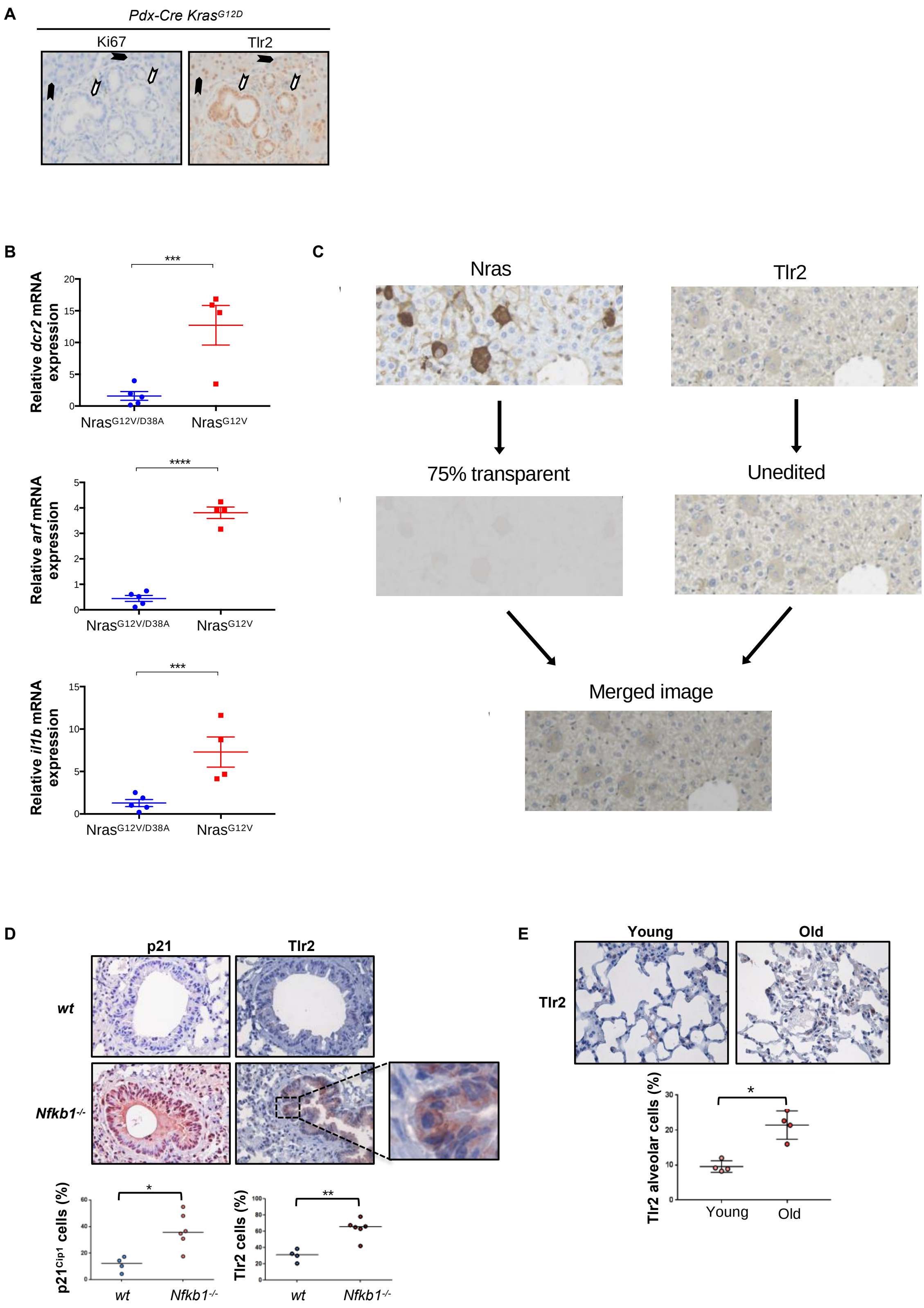

Fig. S2. TLR2 expression is induced during OIS in vivo. (**A**) Immunohistochemical staining of PanIN structures from *Pdx-Cre* $Kras^{G12D}$ for the expression of Tlr2 and Ki67 expression in consecutive sections. White arrows indicate PanIN structure. Black arrows indicate pancreatic acinar cells. (**B**) qRT-PCR analysis of the senescence markers *dcr2* and *arf* and the key SASP factor *il-1β* from snap frozen liver samples from WT mice 6 days after receiving hydrodynamic delivery of $Nras^{G12V/D38A}$ (n=5) and $Nras^{G12V}$ (n=4) transposons respectively. Scatter plot represents value from individual animals and the horizontal line represents group mean ± SEM. Statistics: Students two-tailed *t*-test. ***p<0.001, ****p<0.0001. (**C**) Merged Nras and Tlr2 IHC image. The Nras IHC image was rotated to allow exact alignment with the Tlr2 image, prior to having transparency adjusted to 75%. The resulting image was overlaid with the unedited Tlr2 image to show exact co-expression of Nras and Tlr2 at the cellular level. (**D**) Analysis of Tlr2 and $p21^{Cip-1}$ expression was conducted by immunohistochemistry in lung sections from wild type (wt) or nfkb1 knock out mice ($nfkb1^{-/-}$) at 9.5 months of age. 10-15 random images were captured per mouse and average percentage positivity calculated for airway epithelial compartments. Scatter plots represent mean percentage positivity for each animal with the horizontal line representing group median. Statistical significance was calculated using Mann Whitney U test. *p < 0.05, **p < 0.01. Representative images of p21 and TLR2 staining by immunohistochemistry (positive, brown; negative, blue) in airway epithelial cells from wt and *nfkb1-/-* mice. (**E**) Analysis of Tlr2 expression by immunohistochemistry in lung sections of wt mice at 6.5 months of age (Young) and 24 months of age week (old). Scatter plots were generated from 10-15 random images captured per animal with individual points representing mean percentage positivity for each mouse with horizontal line representing group median. Statistics:

Mann-Whitney U test. *$p < 0.05$. Representative images of TLR2 staining by immunohistochemistry (positive, brown; negative, blue) in alveolar cells from wt mice 6.5 and 24 months of age.

Figure S3

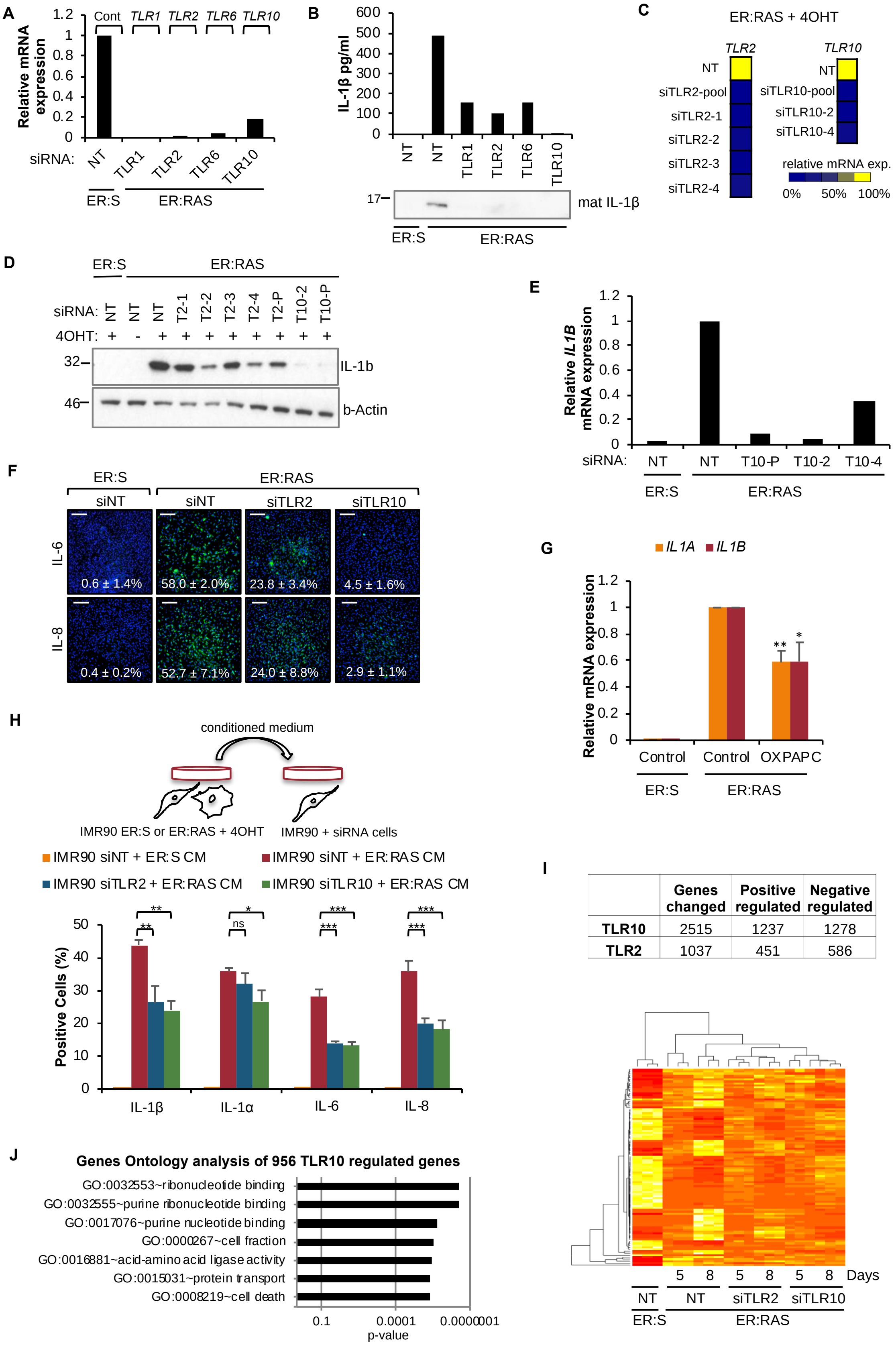

	Genes changed	Positive regulated	Negative regulated
TLR10	2515	1237	1278
TLR2	1037	451	586

Fig. S3. TLR2 and TLR10 regulate the SASP in OIS. (**A-F**) IMR90 ER:RAS cells were treated with 4OHT and repeatedly transfected with indicated pooled siRNA targeting TLR1 subfamily receptors and non-target (NT) siRNA as control for 8 days. (A) qRT-PCR of *TLR1, TLR2, TLR6, TLR10* expression to validate knockdown. (B) ELISA and western blot for IL-1β content in the conditioned medium following indicated TLR knockdown. (C) qPCR of TLR2 and TLR10 expression following the pooled and individual siRNA media knockdown of respective target genes. (D) Western blot of IL-1β protein levels following TLR2 and TLR10 knockdown using pooled (T2-P, T10-P) and individual (T10-2, T2-1, T2-2, T2-3, T2-4) siRNA. (E) qRT-PCR of *IL1B* mRNA expression following TLR10 knockdown using pooled (T10-P) and individual (T10-2, T10-4) siRNA. (F) Representative images of immunofluorescence and high content analysis of IL6, IL8 and IL-1β expression in TLR2 and TLR10 knockdown cells. (**G**) qRT-PCR for IL1A and 1L1B expression in IMR90 ER:RAS cells were treated with 4OHT for 5 days and TLR2 inhibited with 100 μg/ml OxPAPC (**H**) Conditioned medium (CM) generated by IMR90 ER:Stop and ER:RAS following 8 days of 4OHT treatment was transferred to proliferating IMR90 cells with siRNA knockdown of TLR2 and TLR10 and cultured for 2 days. Immunofluorescence and high content analysis was used to determine IL-1α, IL-1β, IL-6 and IL-8 protein levels in the IMR90 cells. Results expressed as mean ± SEM of 3 independent experiments. Statistical significance was calculated using One-Way ANOVA and Dunnett's multiple comparison's tests. ***$p < 0.001$, **$p < 0.01$, * $p < 0.05$, NS, non-significant. (**I**) Transcriptome analysis (Ampliseq) of IMR90 ER:RAS cells transfected with pooled siRNA for TLR2 and TLR10, and non-target (NT) as control (GSE127116). Table identifies the total number of genes significantly

regulated by TLR2 and TLR10 during OIS. Heat-map showing sample clustering. **(J)** Gene ontology analysis on genes regulated by TLR10 in OIS.

Figure S4

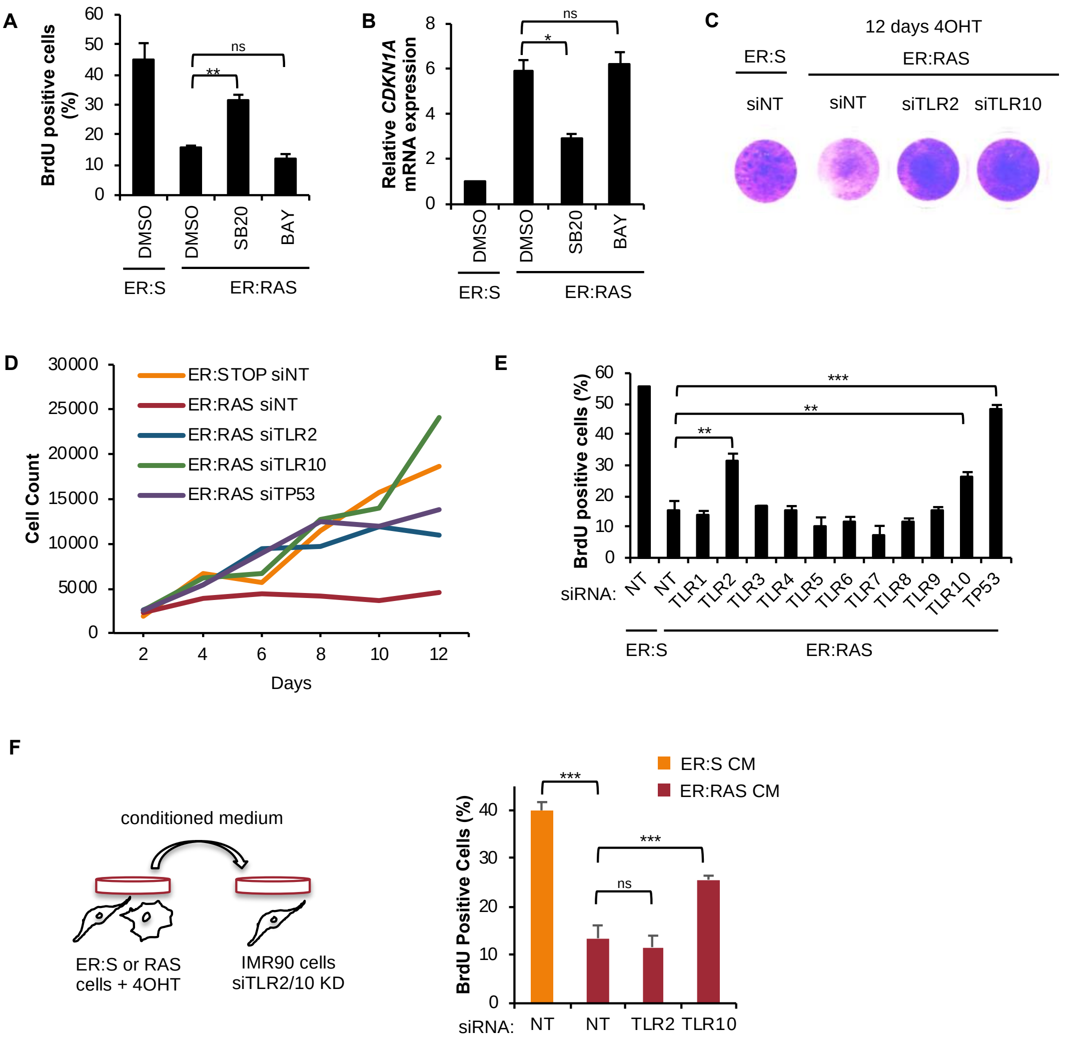
A
BrdU positive cells (%)
60
50
40
30
20
10
0
ns
**
DMSO
DMSO
SB20
BAY
ER:S
ER:RAS
B
Relative CDKN1A mRNA expression
8
6
4
2
0
ns
*
DMSO
DMSO
SB20
BAY
ER:S
ER:RAS
C
12 days 4OHT
ER:S
ER:RAS
siNT
siNT
siTLR2
siTLR10
D
Cell Count
30000
25000
20000
15000
10000
5000
0
ER:STOP siNT
ER:RAS siNT
ER:RAS siTLR2
ER:RAS siTLR10
ER:RAS siTP53
2
4
6
8
10
12
Days
E
BrdU positive cells (%)
60
50
40
30
20
10
0
***
**
**
siRNA:
NT
NT
TLR1
TLR2
TLR3
TLR4
TLR5
TLR6
TLR7
TLR8
TLR9
TLR10
TP53
ER:S
ER:RAS
F
conditioned medium
ER:S or RAS cells + 4OHT
IMR90 cells siTLR2/10 KD
ER:S CM
ER:RAS CM
BrdU Positive Cells (%)
40
30
20
10
0
***
***
ns
siRNA:
NT
NT
TLR2
TLR10

Fig. S4. TLR2 reinforces the cell cycle arrest in OIS. (**A**) IMR90 ER:RAS and ER:Stop cells were treated with 10 μM of IKK inhibitor (BAY 11-07082) and p38 MAPK inhibitor (SB202190). A BrdU incorporation assay was conducted and analysed by high content microscopy. (**B**) *CDKN1A* mRNA expression was measured by qRT-PCR in sample treated as in (A). (**C**) Crystal violet staining of the cells 12 days after siRNA transfection and 4OHT treatment of IMR90 ER:RAS cells. (**D**) Total cell count (nuclei count number) in the total surface of tissue culture wells by high content analysis of IMR90 ER:RAS cells transfected with indicated siRNA for 12 days. (**E**) BrdU incorporation assay following pooled siRNA knockdown of all TLR family members at 5 days of 4OHT treatment in IMR90 ER:RAS and IMR90 ER:Stop cells. Results expressed as mean ± SEM of 3 independent experiments. Statistical significance was calculated using two-tailed Students t-tests. ***$p < 0.001$, **$p < 0.01$, * $p < 0.05$, n.s., non-significant. (**F**) Conditional medium (CM) generated by IMR90 ER:Stop and ER:RAS following 8 days of 4OHT treatment was transferred to proliferating IMR90 cells with siRNA knockdown of TLR2 and TLR10 and cultured for 2 days. Immunofluorescence and high content analysis was used to determine BrdU incorporation in the IMR90 cells. Results expressed as mean ± SEM of 3 independent experiments. Statistical significance was calculated using One-Way ANOVA. ***$p < 0.001$, **$p < 0.01$, * $p < 0.05$, ns, non-significant.

Figure S5

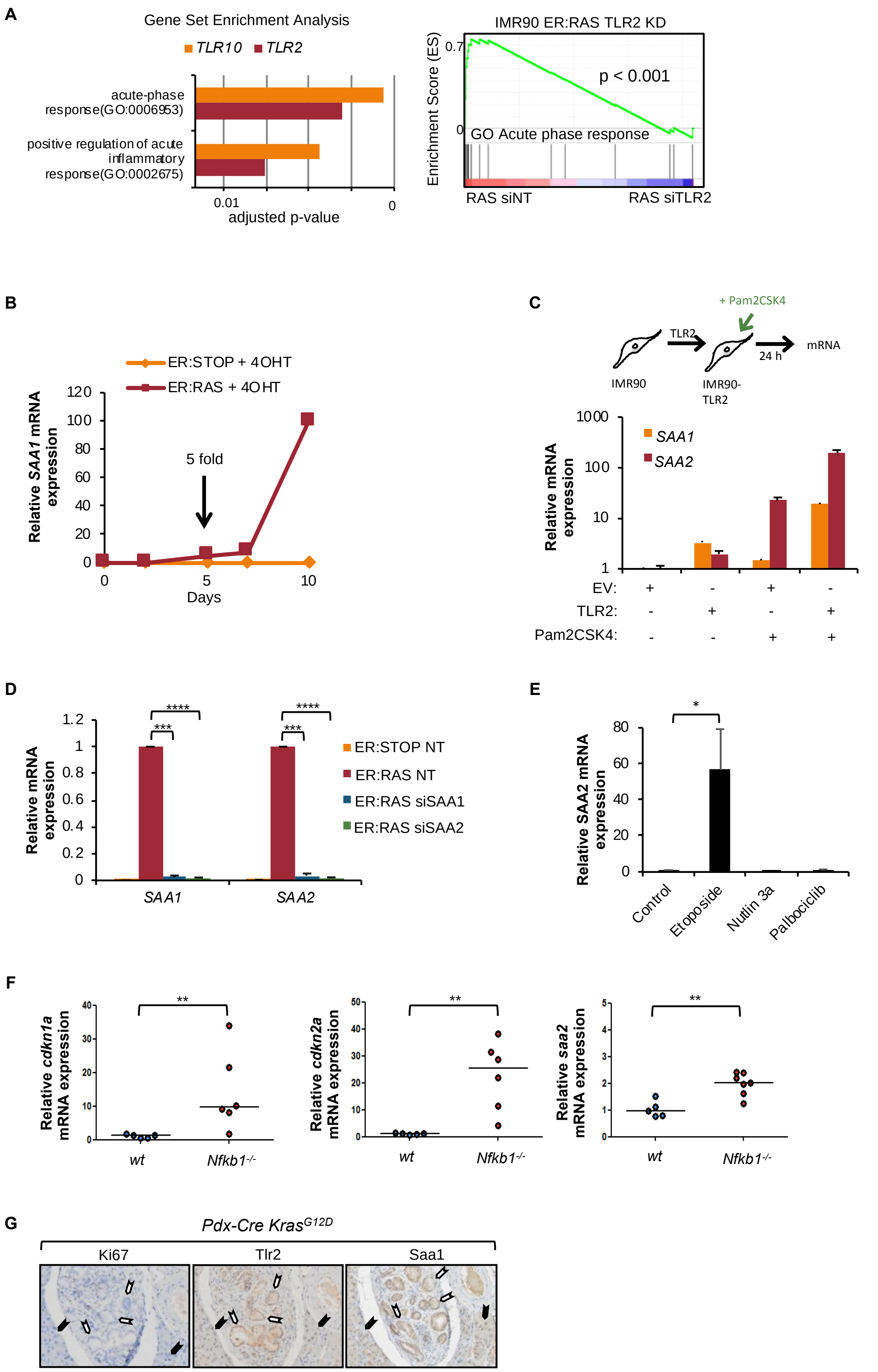
A
Gene Set Enrichment Analysis
TLR10
TLR2
acute-phase response(GO:0006953)
positive regulation of acute inflammatory response(GO:0002675)
0.01
0
adjusted p-value
IMR90 ER:RAS TLR2 KD
Enrichment Score (ES)
0.7
0
p < 0.001
GO Acute phase response
RAS siNT
RAS siTLR2
B
ER:STOP + 4OHT
ER:RAS + 4OHT
Relative SAA1 mRNA expression
5 fold
Days
C
+ Pam2CSK4
TLR2
IMR90
IMR90-TLR2
24 h
mRNA
Relative mRNA expression
SAA1
SAA2
EV:
TLR2:
Pam2CSK4:
D
Relative mRNA expression
ER:STOP NT
ER:RAS NT
ER:RAS siSAA1
ER:RAS siSAA2
SAA1
SAA2
E
Relative SAA2 mRNA expression
Control
Etoposide
Nutlin 3a
Palbociclib
F
Relative cdkn1a mRNA expression
Relative cdkn2a mRNA expression
Relative saa2 mRNA expression
wt
Nfkb1-/-
G
Pdx-Cre KrasG12D
Ki67
Tlr2
Saa1

Fig. S5. TLR2 and TLR10 regulate the activation of genes of the acute-phase response during OIS. (**A**) Gene Set Enrichment Analysis (GSEA) of the transciptome from Supplemental fig. S2H showing top two regulated gene set groups, and GSEA enrichment plot of genes of the acute phase response in TLR2 siRNA transfected IMR90 ER:RAS 4OHT induced cells . (**B**) IMR90 ER:RAS and ER:Stop were treated with 4OHT for up to 10 days with RNA collected at indicated intervals. qRT-PCR for SAA1 mRNA expression (**C**) qRT-PCR for SAA1 and SAA2 mRNA expression in TLR2 overexpressing IMR90 cells activated with 1 µg/ml Pam2CSK4 ligand for 3 hours. Results expressed as mean ± SEM of 3 independent experiments. (**D**) Confirmation of *SAA1* and *SAA2* expression knockdown by qRT-PCR in IMR90 ER:RAS and ER:Stop cells transfected with pooled siRNA targeting SAA1 and SAA2, and non-target (NT) control and treated with 4OHT for 8 days. Results expressed as mean ± SEM of 3 independent experiments. All statistical significance was calculated using One-Way ANOVA. ***$p < 0.001$, **$p < 0.01$, * $p < 0.05$, ns, non-significant. (**E**) qRT-PCR for *SAA2* mRNA expression in IMR90 cells treated as indicated for 7 days. (**F**) qRT-PCR for Cdkn1a (p21), Cdkn2a (p16) and Saa2 expression in whole lung tissue from wt or *nfkb1-/-* mice at 9.5 months of age. Dot plots represent the ΔΔCT value for individual animals generated by normalizing to 18S expression. The horizontal line represents group median. Statistical significance was calculated using Mann-Whitney U test. **$p < 0.01$. (**G**) Immunohistochemical staining of PanIN structures from *Pdx-Cre Kras*^{G12D} for the expression of Tlr2, Saa1 and Ki67 expression in consecutive sections. White arrows indicate PanIN structure. Black arrows indicate pancreatic acinar cells.

Figure S6

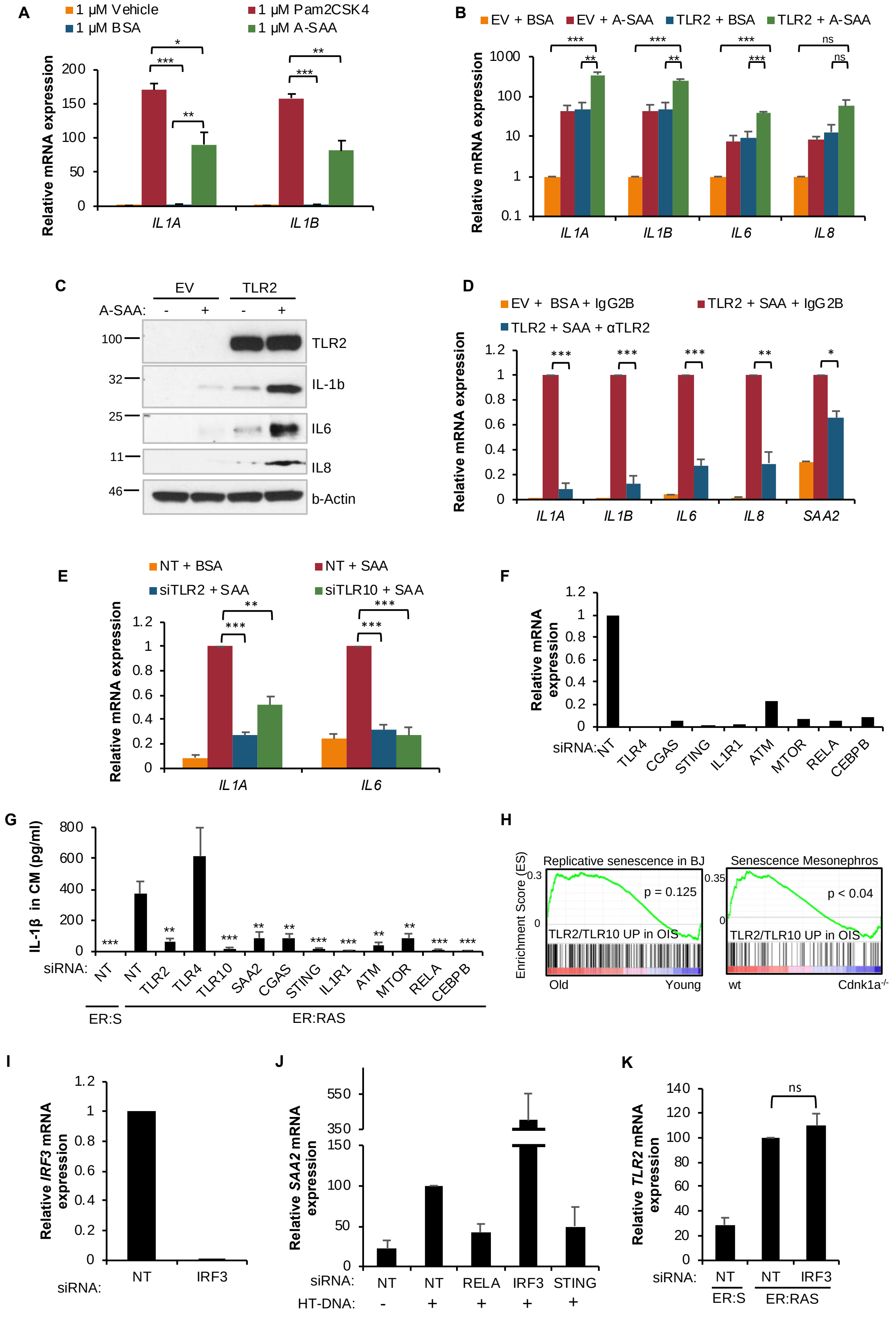
A
1 µM Vehicle
1 µM BSA
1 µM Pam2CSK4
1 µM A-SAA
Relative mRNA expression
IL1A
IL1B
B
EV + BSA
EV + A-SAA
TLR2 + BSA
TLR2 + A-SAA
Relative mRNA expression
IL1A
IL1B
IL6
IL8
C
EV
TLR2
A-SAA:
TLR2
IL-1b
IL6
IL8
b-Actin
D
EV + BSA + IgG2B
TLR2 + SAA + IgG2B
TLR2 + SAA + αTLR2
Relative mRNA expression
IL1A
IL1B
IL6
IL8
SAA2
E
NT + BSA
NT + SAA
siTLR2 + SAA
siTLR10 + SAA
Relative mRNA expression
IL1A
IL6
F
Relative mRNA expression
siRNA:
NT
TLR4
CGAS
STING
IL1R1
ATM
MTOR
RELA
CEBPB
G
IL-1β in CM (pg/ml)
siRNA:
NT
NT
TLR2
TLR4
TLR10
SAA2
CGAS
STING
IL1R1
ATM
MTOR
RELA
CEBPB
ER:S
ER:RAS
H
Enrichment Score (ES)
Replicative senescence in BJ
p = 0.125
TLR2/TLR10 UP in OIS
Old
Young
Senescence Mesonephros
p < 0.04
TLR2/TLR10 UP in OIS
wt
Cdnk1a-/-
I
Relative IRF3 mRNA expression
siRNA:
NT
IRF3
J
Relative SAA2 mRNA expression
siRNA:
NT
NT
RELA
IRF3
STING
HT-DNA:
K
Relative TLR2 mRNA expression
siRNA:
NT
NT
IRF3
ER:S
ER:RAS
ns

Fig. S6. A-SAA signaling through TLR2 controls the SASP. (**A**) *IL1A* and *IL1B* mRNA expression was assessed by qRT-PCR in TLR2 expressing and non-expressing control (EV) IMR90 cells treated with 1 μM A-SAA or 1 μM Pam2CSK4 for 3 hours. Results expressed as mean ± SEM of 3 independent experiments. (**B**) *IL1A, IL1B, IL6* and *IL8* mRNA expression was assessed by qRT-PCR in TLR2 expressing and non-expressing control (EV) IMR90 cells treated with 10 μg/ml A-SAA for 3 hours. Results expressed as mean ± SEM of 3 independent experiments. (**C**) IMR90 cells expressing TLR2 or infected with empty vector (EV) as a control were incubated for 3 hours with recombinant active SAA (A-SAA) protein (10 μg/ml) or BSA (10 μg/ml) as a control. Western blot to measure IL-1β, IL6 and IL8 protein levels as indicated. (**D**) TLR2 overexpressing IMR90 cells were treated with 10 μg/ml A-SAA plus 10 μg/ml of neutralising antibody against TLR2 for 2 hours. Assessment of *IL1A, IL1B, IL6* and *IL8* mRNA expression was conducted by qRT-PCR. Results expressed as mean ± SEM of 3 independent experiments. (**E**) IMR90 cells transfected with pooled siRNA for TLR2 and TLR10 were treated with 1 μg/ml A-SAA for 3 hours and *IL6* and *IL1A* mRNA expression determined by qRT-PCR. Results expressed as mean ± SEM of 3 independent experiments. (**F**) qRT-PCR to confirm the knockdown efficiency of the indicated target gene by pooled siRNA. (**G**) IMR90 ER:RAS cells were treated with 4OHT and repeatedly transfected with indicated pooled siRNA and non-target (NT) siRNA as control for 8 days. ELISA for IL-1β levels in conditioned medium. Results expressed as mean ± SEM of 3 independent experiments. (**H**) GSEA plots of the gene set from Supplemental fig. S4A in the transcriptome of replicative senescence BJ cells and programmed senescent cells of the mesonephros in Fig. 4I. (**I**) IMR90 cells were transfected with siRNA targeting RELA, IRF3, TLR2 and STING for 2 days followed by transfection with 2.5 μg herrings-testes DNA (HT-

DNA) for 24 hours. *IRF3 and SAA2* transcripts were measured by qRT-PCR. Results are expressed as mean ± SEM of 3 independent experiments. (**J**) qRT-PCR of *TLR2* expression in IMR90 ER:RAS cells with siRNA knockdown of IRF3 for 8 days. IMR90 ES:S cells and NT siRNA as control. All statistical significance was calculated using One-Way ANOVA. ***$p < 0.001$, **$p < 0.01$, * $p < 0.05$, ns, non-significant.

Figure S7

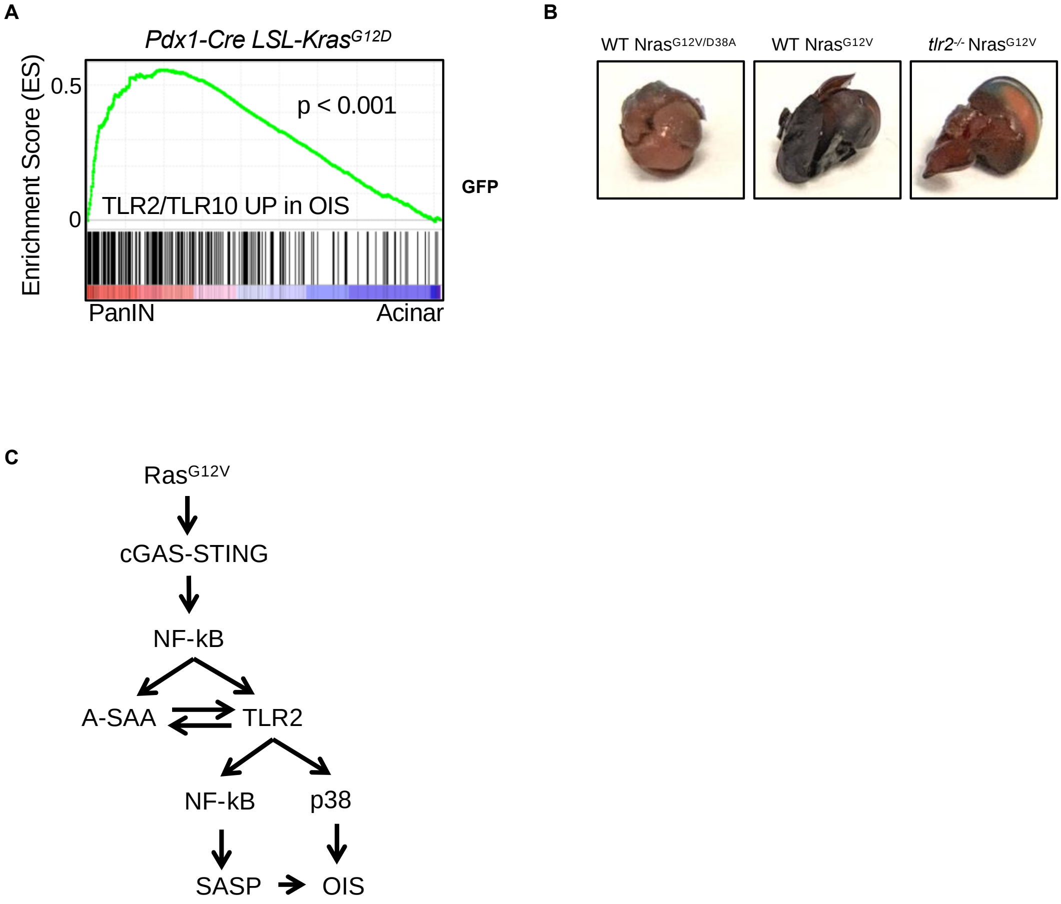
A
Pdx1-Cre LSL-KrasG12D
Enrichment Score (ES)
0.5
0
p < 0.001
TLR2/TLR10 UP in OIS
PanIN
Acinar
GFP
B
WT NrasG12V/D38A
WT NrasG12V
tlr2-/- NrasG12V
C
RasG12V
cGAS-STING
NF-kB
A-SAA
TLR2
NF-kB
p38
SASP
OIS

Fig. S7. *tlr2* is necessary for OIS activation in vivo. (**A**) GSEA enrichment plot of genes regulated by TLR2 and TLR10 in OIS described in Supplemental fig. S2G in the transcriptome of PanIN cells compared to acinar normal cells (GSE33323). (**B**) Whole mount SA-B-Gal staining of liver tissue from mice described in Fig 6B. (**C**) Model of activation of the SASP by A-SAA-TLR2 in OIS. (Photo Credit: Juan Carlos Acosta, Cancer Research UK- Edinburgh Centre).

Table S1. Primers used for qRT-PCR in this study.

Target	Forward primer	Reverse primer
Actin	CATGTACGTTGCTATCCAGGC	CTCCTTAATGTCACGCACGAT
INK4A	CGGTCGGAGGCCGATCCAG	GCGCCGTGGAGCAGCAGCAGCT
CDKN1A	CCTGTCACTGTCTTGTACCCT	GCGTTTGGAGTGGTAGAAATCT
IL8	GAGTGGACCACACTGCGCCA	TCCACAACCCTCTGCACCCAGT
IL6	CCAGGAGCCCAGCTATGAAC	CCCAGGGAGAAGGCAACTG
CCL20	GGCGAATCAGAAGCAGCAAGCAAC	ATTGGCCAGCTGCCGTGTGAA
INHBA	CGGCGCTTCTGAACGCGATC	GCTGTTCCTGACTCGGCAAACGT
IL1A	AGTGCTGCTGAAGGAGATGCCTGA	CCCCTGCCAAGCACACCCAGTA
IL1B	TGCACGCTCCGGGACTCACA	CATGGAGAACACCACTTGTTGCTCC
TLR1	GTTTTTGTGGCCAGGGTCTTC	TAGGGGTGCCCAATATGCCT
TLR2	CTCGGAGTTCTCCCAGTGTT	TGGCATTGTCCAGTGCTTCA
TLR3	AGTGCCGTCTATTTGCCACA	TGATTCTGTTGGATGACTGCT
TLR4	GGTCAGACGGTGATAGCGAG	TTAGGAACCACCTCCACGCA
TLR5	TGATGTTCATGTTCCTGACACT	AGCATCCCTGGTTTGGTGAC
TLR6	TGCATTAGCCCTTCCTTGCTC	TGTGGAAGAATGTGCCGTTTG
TLR7	GGCCCATCTCAAGCTGATCT	GTCCACATTGGAAACACCATTT
TLR8	GAAACATGGTTCTCTTGACACTTCA	TCCTTCAAGGTCTCCTGGGAT
TLR9	CTTCCCTGTAGCTGCTGTCC	CAGAAACCCATGCTGGGGG
TLR10	ATGCTAGTTCTGGGGTTGGC	CCCTGTGCCATGTTTGTGTG
SAA1	GAGCACACCAAGGAGTGATTT	GAAGCTTCATGGTGCTCTCT
SAA2	GCTGCAGAAGTGATCAGCAAT	CAGCGAGTCCTCCGCAC
IL6R	TCACTGTGTCATCCACGACG	CTGGATTCTGTCCAAGGCGT
SERPINA3	TAGCAGTCTCCCAGGTGGTC	GGCCTGTTGAAACGCACAAT
PTGS2	CAAATTGCTGGCAGGGTTGC	AGGGCTTCAGCATAAAGCGT
STAT3	GAAACAGTTGGGACCCCTGA	AGGTACCGTGTGTCAAGCTG
STAT5B	ACATTAAGGCCACCCAGCTC	AGCGGTCATACGTGTTCTGG
CEBPB	TTTGTCCAAACCAACCGCAC	GCATCAACTTCGAAACCGGC
TGFB1	CACCGGAGTTGTGCGGCAGT	GGCCGGTAGTGAACCCGTTGATG
MMP1	AAAGGGAATAAGTACTGGGC	CAGTGTTTTCCTCAGAAAGAG
MMP3	TAAAGACAGGCACTTTTGG	GAGATGGCCAAAATGAAGAG
MTOR	TCGCTGAAGTCACACAGACC	CTTTGGCATATGCTCGGCAC
RELA	CCAGACCAACAACAACCCCT	TTGGGGGCACGATTGTCAAA
CEBPB	TTTGTCCAAACCAACCGCAC	GCATCAACTTCGAAACCGGC
CGAS	AAGAAGAGAAATGTTGCAGGAAAG	GACTGTCTTGAGGGTTCTGGG
STING	ATATCTGCGGCTGATCCTGC	TTGTAAGTTCGAATCCGGGC
IL1R1	ACGTTGGGGAAGACATTGTTGAGG	ACCCAGCCAGCTGAAGCCTGA
ATM	TTGAACCGGAAGCGGGAGTA	GATGTATGCCTCACTGCACTC
IRF3	ACCTGGGGCCCTTCATTGTA	GCACAACCTTGACCATCACG
β-actin	GGCACCACACCTTCTACAA	GTGGTGGTGAAGCTGTAGCG
Tlr2	CTAGAAGTGGAAAAGATGTCG	TAGCATCCTCTGAGATTTGAC
Arf	GCCGCACCGGAATCCT	TTGAGCAGAAGAGCTGCTACGT
Dcr2	AGCTAACCCAGCCCATAATCGTC	AGTTCCCTTCTGACAGGTACTGGC
Il1a	AGGAGAGCCGGGTGACAGTA	TCAGAATCTTCCCGTTGCTTG
Il1b	CCAAAAGATGAAGGGCTGCT	TCATCAGGACAGCCCAGGTC
Il6	CAAGAAAGACAAAGCCAGAGTC	GAAATTGGGGTAGGAAGGAC
Cdkn2a	TTGCCCATCATCATCACCT	GGGTTTTCTTGGTGAAGTTCG
Cdkn1a	CCTGGTGATGTCCGACCTG	CCATGAGCGCATCGCAATC
Saa2	GCCTTCTTAGACAGAGCAGGA	CCTTGGAAAGCCTCCCCAATA
18s	CGGCTACCACATCCAAGGAA	AGCCGCGGTAATTCCAGC

Table S2. siRNA sequences used in this study. All ON-TARGETplus siRNA (Dharmacon).
SMARTpool

Gene Symbol	Catalogue Number
TLR1	L-008086-00
TLR2	L-005120-01
TLR3	L-007745-00
TLR4	L-008088-01
TLR5	L-008089-00
TLR6	L-005156-00
TLR7	L-004714-00
TLR8	L-004715-00
TLR9	L-004066-00
TLR10	L-008087-00
SAA1	L-019361-00
SAA2	L-016279-02
MTOR	L-003008-00
RELA	L-003533-00
IL1R1	L-005188-00
ATM	L-003201-00
MB21D1 (cGAS)	L-015607-02
TMEM173 (STING)	L-024333-02
CEBPB	L-006423-00
TP53	L-016930-00
IRF3	L-006875-00
Non-targeting	D-001810-10

Individual siRNA

Gene Symbol	Catalogue Number	Sequence
siTLR2-1	J-005120-14	AAAUCUGAGAGCUGCGAUA
siTLR2-2	J-005120-15	AGGUAAAGUGGAAACGUUA
siTLR2-3	J-005120-16	UGUUUGGAACUGCGAGAUA
siTLR2-4	J-005120-17	AGUAGGAAUGCAAUAACUA
siTLR10-2	J-008087-06	CGAAUUAUCUUGCAACACA
siTLR10-4	J-008087-08	CAUCGGUUCUAUUGCUUAA

Table S3. Antibodies used in this study.

REAGENT or RESOURCE	SOURCE	IDENTIFIER	USAGE
Primary Antibodies			
Mouse monoclonal anti-IL1A	R&D systems	Cat #MAB200	WB 1:1000, IF 1:100
Mouse monoclonal anti-IL1B	R&D systems	Cat #MAB201	WB 1:200 - 1:5000, IF 1:100
Goat polyclonal anti-IL6	R&D systems	Cat #AF206NA	WB 1:1000, IF 1:100
Mouse monoclonal anti-IL8	R&D systems	Cat #MAB208	WB 1:1000, IF 1:100
Mouse monoclonal anti-p53 (DO1)	Santa Cruz	Cat #sc-126	WB 1:1000, IF 1:100
Rabbit monoclonal anti-TLR2 [EPNCIR133]	Abcam	Cat #ab108998	WB 1:1000, IF 1:100
Rabbit polyclonal anti-TLR10	Santa Cruz	Cat #sc-30198	WB 1:1000
Mouse monoclonal anti-BrdU	BD Phamingene	Cat #555627	IF 1:2000
Rabbit polyclonal anti-IKKα	CST	Cat #2682	WB 1:1000
Rabbit polyclonal anti-IKKβ (L570)	CST	Cat #2678	WB 1:1000
Rabbit monoclonal anti-IKKα/β (Ser176/180) (16A6)	CST	Cat #2697	WB 1:1000
Rabbit monoclonal anti-p38 MAPK (D13E1) XP	CST	Cat #8690	WB 1:5000
Rabbit polyclonal anti-phospho-p38 MAPK (Thr180/Tyr182)	CST	Cat #9211	WB 1:1000
Rabbit monoclonal anti-STING (D2P2F)	CST	Cat #13647	WB 1:1000
Rabbit monoclonal anti-phospho NF-kB p65 (Ser536)	CST	Cat #3033	WB 1:500
Mouse monoclonal anti-NF-kB p65 (F-6)	Santa Cruz	Cat #sc-8008	WB 1:1000
Mouse monoclonal anti-TLR2	R&D systems	Cat #MAB2616	Neut 10 µg/ml
Mouse monoclonal IgG2B Isotype Controls (20116)	R&D systems	Cat #MAB004	Neut 10 µg/ml
Rabbit monoclonal anti-Ki67 [SP6]	Abcam	Cat #ab21700	IHC
Rabbit polyclonal anti-TLR2	Invitrogen	Cat #PA5-20020	IHC 1:1000
Rabbit polyclonal anti-SAA1	Biorbyt	Cat #orb228668	IHC 1:200
Rabbit polyclonal anti-p21	Abcam	Cat #ab7960	IHC 1:200
Mouse monoclonal anti-N-Ras (F155)	Santa Cruz	Cat #sc-31	IHC 1:300
Rabbit polyclonal anti-IL-1b (H-153)	Santa Cruz	Cat #sc-7884	IHC 1:100
Rabbit polyclonal anti-GFP	Abcam	Cat#ab6556	IHC 1:1000
Goat polyclonal anti-IL1A	R&D systems	Cat#AF-400	IHC 1:200
Mouse anti p21	BD Pharmingene	Cat #556431	IHC 1:50
Mouse monoclonal anti-Biotin	Abcam	Cat #ab201341	IHC 1:100
Seconary Antibodies			
Goat anti-Mouse IgG Alexa Fluor 488	Thermo Fisher	Cat #A11029	IF 1:1000
Donkey anti-Goat IgG Alexa Fluor 594	Thermo Fisher	Cat #A-11058	IF 1:1000
Anti-Mouse IgG (Fc specific)-Peroxidase	Sigma	Cat #A2554	WB 1:20,000 - 1:80,000
Anti-Rabbit IgG (whole molecule)-Peroxidase	Sigma	Cat #A0545	WB 1:20,000 - 1:80,000
Donkey anti-goat-HRP	Santa Cruz	Cat #sc-2020	WB 1:20,000
Goat anti-Rabbit IgG Alexa Fluor 594	Thermo Fisher	Cat #A11037	IF 1:1000
Monoclonal anti-B-Actin-peroxidase (AC-15)	Sigma	Cat #A3854	WB 1:100,000 - 1:1,000,000
Horse anti-mouse IgG HRP	Vactor Labs	Cat #PI-2000	IHC 1:100
Biotinylated Goat anti-Mouse IgG	Vactor Labs	Cat #BA9200	IHC 1:100
Biotinylated Goat anti-Rabbit IgG	Vactor Labs	Cat #BA1000	IHC 1:100
